# Analysis of the global burden and key risk factors of neonatal sepsis and other neonatal infections in 204 countries and territories, 1990–2021

**DOI:** 10.3389/fmed.2025.1536948

**Published:** 2025-04-04

**Authors:** Caini Mu, Feng Liu, Tian Tian, Miaona Feng, Xinran Dang, Luyin Xie, Jianzhou Liu, Xuan Li

**Affiliations:** ^1^Department of Infection Control, Xi’an International Medical Center Hospital, Xi’an, China; ^2^Department of Epidemiology, School of Public Health, China Medical University, Shenyang, China; ^3^Department of Pediatrics, Xi’an International Medical Center Hospital, Xi’an, China; ^4^Department of Medical Affairs, Xi’an International Medical Center Hospital, Xi’an, China

**Keywords:** neonatal infections, sepsis, global disease burden, key risk factors, other neonatal infections

## Abstract

**Background:**

Neonatal infections, particularly neonatal sepsis, remain significant contributors to morbidity and mortality in pediatrics. This study aims to provide data support for health authorities to control neonatal infections by analyzing the burden of neonatal sepsis and other neonatal infectious (NSNIs) globally and the trends in their risk factors.

**Methods:**

This study is based on the Global Burden of Disease (GBD) database, reviewing the burden and trends of neonatal sepsis and other infectious diseases from 1990 to 2021 at global, regional, and national levels. Descriptive statistics and Joinpoint regression analyses were employed to assess incidence rates, prevalence rates, mortality rates, and Disability-Adjusted Life Years (DALYs), with the Average Annual Percent Change (AAPC) used to quantify these trends.

**Results:**

The findings reveal that from 1990 to 2021, the global incidence (AAPC = −0.8%, *p* < 0.01), prevalence (AAPC = −0.8%, *p* < 0.01), DALYs (AAPC = −0.9%, *p* < 0.01), and mortality rates (AAPC = −0.9%, *p* < 0.01) for neonatal sepsis and other neonatal infections showed a downward trend. The burden was notably higher in males than in females. Regional analysis indicated that the disease burden remains high in Africa and Southeast Asia, with DALYs of 367,540.10/100,000 and 180,599.79/100,000, respectively. Conversely, the burden in the Eastern Mediterranean and Western Pacific regions has been rising, with DALYs increasing from 53,165.45/100,000 in 2016 to 57,179.59/100,000 in 2021, and from 125,896.44/100,000 in 2018 to 131,698.77/100,000 in 2021. National-level data revealed that Sierra Leone, Chad, and Burkina Faso had significantly higher burdens compared to other countries, with DALYs of 534,090.25/100,000, 520,317.08/100,000, and 505,365.73/100,000 in 2021. An analysis of risk factors indicated that DALYs associated with ambient particulate matter pollution increased by 0.7% since 1990, while DALYs from Household air pollution in solid fuels decreased by 1.4%. Although the burden of diseases related to low birth weight and short gestation declined in many countries, an upward trend was observed in the Eastern Mediterranean and Western Pacific regions (DALYs increased from 88,653.41/100,000 in 2018 to 93,752.24/100,000 in the Eastern Mediterranean and from 28,813.84/100,000 in 2017 to 32,280.55/100,000 in the Western Pacific).

**Conclusion:**

The analysis indicates that while the global burden of NSNIs has decreased, the situation remains serious in Africa and Southeast Asia, with a continuing rise in the burden of NSNIs in the Eastern Mediterranean and Western Pacific regions in recent years. Policymakers should prioritize improving healthcare facilities, increasing access to medical resources, and investing in maternal and neonatal care to effectively reduce the incidence of NSNIs.

## 1 Introduction

Neonatal infections are one of the leading causes of mortality among newborns globally, particularly in low- and middle-income countries (1, 2). A neonatal infection is defined as an infection that occurs within the first 28 days after birth, primarily caused by maternal infections, environmental exposures, or postnatal contact. These infections can be transmitted through vertical transmission, infections during delivery, or postnatal contact. Common pathogens include Group B *Streptococcus, Escherichia coli*, and herpes simplex virus (3, 4). The neonatal immune system is relatively fragile, especially in preterm infants, which significantly increases their risk of infection (5).

Ambient particulate matter pollution, low birth weight, and short gestation are critical risk factors influencing neonatal sepsis and other neonatal infections. Environmental particulate matter can enter the uterus through maternal inhalation or be transmitted during delivery. Research indicates that there is a positive correlation between air pollution levels and the incidence of neonatal sepsis, which may adversely affect maternal health, leading to short gestation and low birth weight, thus increasing the risk of neonatal infections (6). Infants with low birth weight have an immature immune system, making them more susceptible to infections and associated with higher rates of perinatal mortality, indicating a greater likelihood of experiencing infections or other serious health issues (7). Similarly, short gestation is a significant risk factor, as the immune systems of preterm infants are underdeveloped, rendering them more vulnerable to infections (8). Furthermore, short gestation is closely associated with maternal nutritional status and health, which further increases the risk of infections during delivery (9).

According to the Global Burden of Disease (GBD) data (10), approximately 500,000 neonates died from infections in 2019. Although this number has declined since 2015, neonatal infections remain a major cause of mortality, with around 200,000 newborns succumbing to these infections each year (11). The burden of infection is particularly severe in South Asia and sub-Saharan Africa. A hospital report from South Asia noted that there are 15.8 cases of infectious shock for every 1,000 neonates (12). Therefore, early identification and timely treatment are crucial in controlling neonatal infections.

To effectively mitigate the impact of neonatal infections, a comprehensive assessment of disease burden, trends, and associated risk factors across different regions and countries is necessary. This study aims to utilize data from the GBD database spanning from 1990 to 2021 to review and analyze the global, regional, and national trends of neonatal sepsis and other neonatal infections (NSNIs). By thoroughly investigating the burden of neonatal infections, this research will reveal disparities in infection burden across various socio-economic backgrounds and geographical regions and evaluate the impact of key influencing factors on neonatal health.

In conclusion, ambient particulate matter pollution, low birth weight, and short gestation interact as multiple risk factors, exacerbating the burden of neonatal infections. The findings of this study aim to provide scientific evidence for policymakers to develop more targeted public health intervention strategies, ultimately improving neonatal survival and health outcomes.

## 2 Methods

### 2.1 Data source

This study utilized data from the GBD database, which covers information related to NSNIs from 1990 to 2021 (13, 14). The GBD database, established by the Institute for Health Metrics and Evaluation at the University of Washington, aggregates high-quality health data from countries worldwide to assess the impact of various diseases on global and regional health. The GBD study annually recalibrates its data to ensure the provision of the latest health statistics and the most accurate analytical results (15).

### 2.2 Study design

This retrospective ecological study aimed to analyze the burden trends of NSNIs from 1990 to 2021. We compared data from different time points to explore changes and regional disparities over the study period, encompassing classifications for both neonatal sepsis and all related neonatal infections.

The primary indicators included incidence rates, prevalence rates, mortality rates, and Disability-Adjusted Life Years (DALYs). The latter refers to the years of healthy life lost due to disability. DALYs represent the weighted combination of Years Lived with Disability and Years of Life Lost (16, 17).

The time span of the study extends from 1990 to 2021, with regions classified according to the World Health Organization (WHO) as the Americas, Eastern Mediterranean, Europe, Western Pacific, Southeast Asia, and Africa (18). Additionally, to assess the socioeconomic development levels of countries or regions, the study utilized the Socio-Demographic Index (SDI) (18), which considers per capita gross national income educational attainment, and total fertility rate, categorizing it into five levels: high, high-middle, middle, low-middle, and low (19).

### 2.3 Statistical analysis

This study focused on NSNIs-related data from 204 countries and regions, employing descriptive statistics to reflect trends in the disease burden and calculate estimates with 95% uncertainty intervals (UI). The estimates provided by the GBD account for variance in parameters and uncertainties related to data collection and model selection (20).

Trend analysis utilized Joinpoint regression software (version 4.9.1.0), calculating the Average Annual Percent Change (AAPC) for NSNIs from 1990 to 2021. This method identifies significant joinpoints, segmenting trends into different phases and calculating the Annual Percent Change (APC) for each stage alongside its 95% UI (21). An APC greater than zero indicates an upward trend, while a negative APC indicates a downward trend, with significance evaluated by *p*-values (*p* < 0.05 considered significant) (22).

Moreover, the analysis evaluated the impacts of key risk factors related to NSNIs (such as ambient particulate matter pollution, low birth weight, and short gestation) (23) on disease burden, employing R software (version 4.4.1) for data visualization of trends in incidence rates, prevalence rates, DALYs, and mortality rates for NSNIs at global, regional, and national levels. The GBD study associates “short gestation” with preterm birth, defined as delivery before 37 weeks. It is assessed in relation to neonatal health risks, the health status of preterm infants, and the disease burden (24, 25).

## 3 Results

### 3.1 Global trends of neonatal sepsis and other neonatal infections (NSNIs) from 1990 to 2021

**Figure 1:**
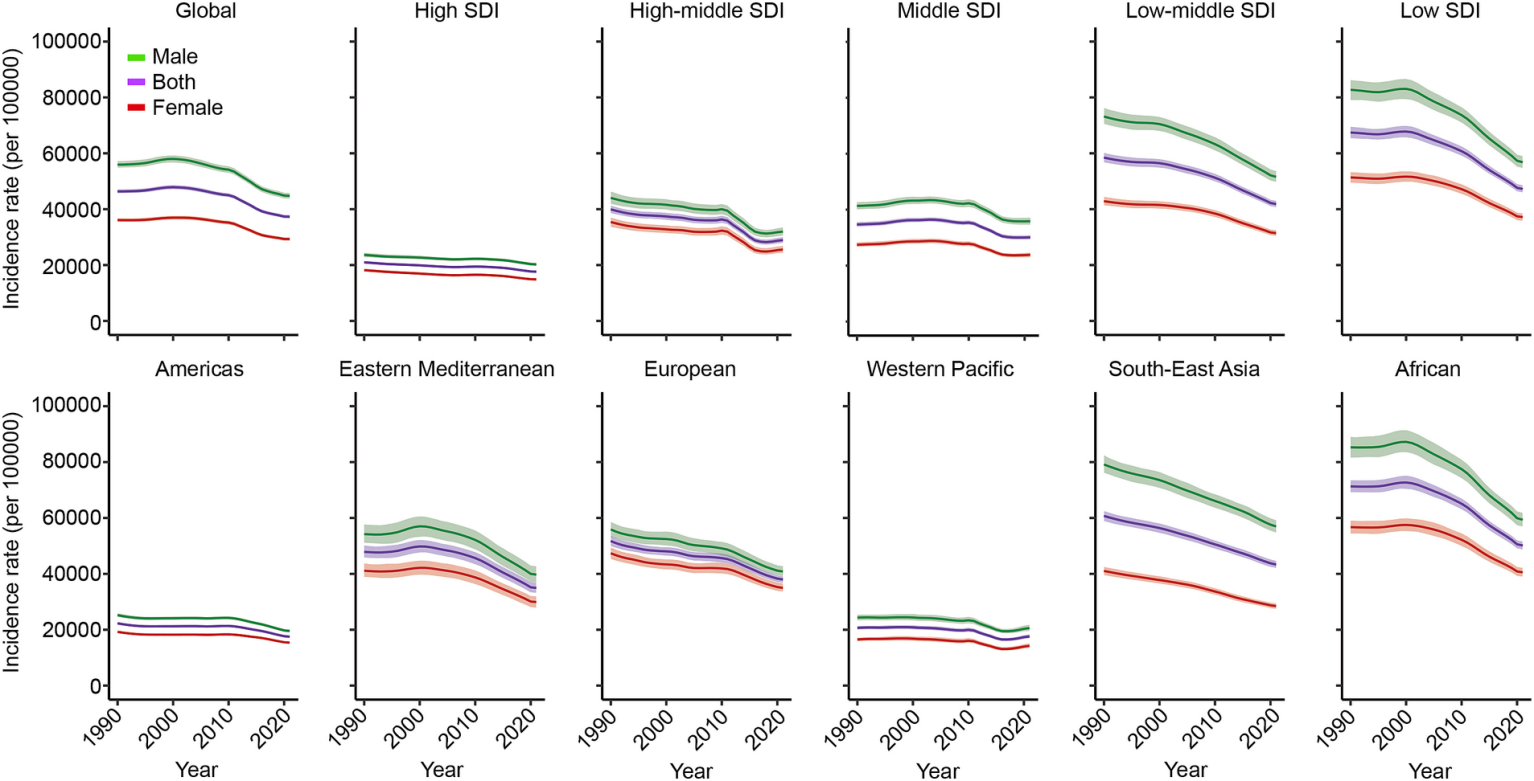
Trends of incidence rate of NSNIs at the global and regions from 1990 to 2021. NSNIs: neonatal sepsis and other neonatal infections.

Globally, the incidence of NSNIs decreased from 46,365.63/100,000 in 1990 to 37,294.43/100,000 in 2021 (AAPC = −0.8%, *p* < 0.01). The prevalence also fell from 598.08/100,000 in 1990 to 465.57/100,000 in 2021 (AAPC = −0.8%, p < 0.01). DALYs decreased from 238,416.27/100,000 in 1990 to 190,657.60/100,000 in 2021 (AAPC = −0.9%, *p* < 0.01), while the mortality rate declined from 2,648.99/100,000 in 1990 to 2,118.49/100,000 in 2021(AAPC = −0.9%, p < 0.01). The trends among males and females were similar, although the incidence rate was higher in males. Overall, from 1990 to 2021, the incidence and mortality rates of NSNIs showed a general declining trend worldwide. However, in recent years, there has been a notable adverse trend in the Western Pacific region, where the incidence rate increased from 16,489.15 per 100,000 in 2017 to 17,608.97 per 100,000 in 2021, and the mortality rate rose from 586.92 per 100,000 in 2016 to 635.23 per 100,000 in 2021. Similarly, in the Eastern Mediterranean region, the mortality rate increased from 1,398.68 per 100,000 in 2018 to 1,463.22 per 100,000 in 2021 (Figure 1, Supplementary Figures S1–S3, Table 1).

**Table 1:** Trends in the burden of neonatal sepsis and other neonatal infections globally and regionally from 1990 to 2021 (per 100,000).

Regions	Incidence rate			Prevalence rate			DALYs rate			Death rate		
	1990 (95%UI)	2021 (95%UI)	AAPC	1990 (95%UI)	2021 (95%UI)	AAPC	1990 (95%UI)	2021 (95%UI)	AAPC	1990 (95%UI)	2021 (95%UI)	AAPC
Global	46365.63 (45720.759, 47080.005)	37294.434 (36742.274, 37915.165)	−0.8	598.077 (424.852, 834.256)	465.571 (365.167, 602.41)	−0.8	238416.27 (210446.838, 265861.961)	190657.6 (161129.49, 224146.627)	−0.9	2648.993 (2337.831, 2953.867)	2118.49 (1790.393, 2490.714)	−0.9
High SDI	20978.716 (20550.698, 21452.546)	17621.971 (17315.654, 17937.981)	−0.4	289.248 (216.309, 387.686)	236.442 (189.425, 300.999)	−0.5	30810.054 (27660.359, 34528.896)	14038.304 (12386.934, 15556.5)	−2.8	342.067 (306.926, 383.415)	155.776 (137.445, 172.65)	−2.8
High-middle SDI	39920.957 (38599.642, 41275.068)	28920.432 (27952.855, 29925.588)	−1.1	530.071 (385.065, 718.079)	378.611 (300.741, 484.409)	−1.1	65420.705 (57992.65, 73322.98)	34296.026 (29133.468, 39971.304)	−2.4	726.371 (643.723, 813.796)	380.778 (323.375, 443.812)	−2.4
Middle SDI	34691.273 (33915.05, 35522.259)	30111.954 (29367.125, 30842.149)	−0.5	448.917 (322.548, 619.794)	382.344 (299.203, 491.947)	−0.6	147977.628 (127616.987, 172488.862)	109752.871 (90593.928, 133588.595)	−1.2	1644.008 (1417.887, 1916.554)	1219.386 (1006.514, 1484.331)	−1.2
Low-middle SDI	58459.896 (56805.514, 60132.325)	41836.527 (40745.69, 42942.435)	−1.1	750.392 (523.876, 1063.78)	519.798 (405.53, 667.694)	−1.1	348555.327 (300930.352, 400719.229)	210808.301 (170917.501, 259712.9)	−1.7	3872.908 (3343.756, 4452.349)	2342.392 (1899.171, 2885.903)	−1.7
Low SDI	67431.595 (65432.735, 69469.925)	47243.06 (46033.556, 48552.485)	−1.1	850.097 (593.411, 1216.113)	576.142 (444.772, 749.604)	−1.2	482534.033 (421173.967, 551486.224)	343708.076 (277975.551, 423698.816)	−1.0	5361.85 (4679.876, 6127.373)	3819.39 (3088.969, 4708.461)	−1.0
Americas	22281.248 (21900.623, 22683.099)	17512.135 (17236.945, 17803.335)	−0.5	292.838 (212.595, 399.027)	225.168 (177.179, 289.66)	−0.6	171864.702 (159718.84, 185504.369)	103016.42 (82065.742, 125631.957)	−1.6	1909.691 (1774.612, 2061.114)	1144.672 (911.841, 1396.03)	−1.6
European	47863.675 (45881.804, 50140.446)	34965.464 (33253.029, 36881.244)	−1.0	616.997 (428.923, 867.493)	434.207 (335.311, 566.551)	−1.0	151092.702 (121449.921, 189657.509)	131698.767 (101946.841, 164956.965)	−0.8	1678.338 (1348.988, 2107.172)	1463.218 (1132.559, 1832.862)	−0.8
Eastern Mediterranean	51725.193 (49952.525, 53562.293)	38003.222 (36853.03, 39115.27)	−0.9	695.1 (513.216, 938.793)	499.615 (397.931, 637.411)	−0.9	55244.457 (48363.584, 63440.904)	32898.373 (28407.586, 37991.795)	−1.7	613.137 (536.228, 704.467)	365.126 (315.124, 421.746)	−1.7
Western Pacific	20671.638 (20074.281, 21298.85)	17608.967 (16954.317, 18344.523)	−0.8	269.425 (194.592, 370.26)	225.27 (175.672, 290.252)	−0.8	79573.43 (67402.795, 92759.458)	57179.586 (48050.587, 67524.519)	−1.5	883.966 (748.822, 1030.662)	635.234 (533.709, 750.179)	−1.5
South-East Asia	60712.485 (58917.701, 62436.694)	43330.832 (42150.865, 44590.38)	−1.1	779.424 (543.463, 1101.689)	543.094 (423.93, 694.761)	−1.1	324915.372 (279485.185, 378239.612)	180599.793 (144573.244, 220468.12)	−2.0	3610.178 (3105.154, 4203.041)	2006.665 (1606.324, 2449.72)	−2.0
African	71292.173 (69237.458, 73475.626)	50143.032 (48733.947, 51524.93)	−1.2	902.066 (633.61, 1285.382)	613.181 (473.514, 797.903)	−1.2	520318.567 (446182.668, 587634.89)	367540.097 (302629.912, 446330.433)	−1.0	5781.723 (4957.8, 6530.044)	4084.214 (3362.726, 4960.088)	−1.0

AAPC, average annual percentage change; DALY, disability-adjusted life year; SDI, sociodemographic index; UI, uncertainty interval.

### 3.2 Regional trends of neonatal sepsis and other neonatal infections (NSNIs) from 1990 to 2021

From 1990 to 2021, regions with lower SDI experienced a period of stability in NSNIs incidence, followed by a slight increase (from 66,802.65/100,000 in 1995 to 67,782.42/100,000 in 2000), but a notable decrease recently. In contrast, the incidence rate in regions with a middle-high SDI exhibited a slow decline followed by a recent increase (from 28,361.03/100,000 in 2017 to 28,920.43/100,000 in 2021). The Eastern Mediterranean and Africa also showed a stable period followed by slight increases (Eastern Mediterranean from 47,654.45/100,000 in 1993 to 49,809.36/100,000 in 2000; Africa from 71,209.36/100,000 in 1992 to 72,687.09/100,000 in 2000), followed by significant decreases in recent years. The Western Pacific region displayed a trend of gradual decline followed by an increase in recent years (from 16,489.15/100,000 in 2017 to 17,608.97/100,000 in 2021) (Figure 1, Supplementary Figures S1–S3, Supplementary Tables S1–S4).

The research indicates that the infection burden remains high in the Africa and Southeast Asia regions (DALYs of 367,540.10/100,000 and 180,599.79/100,000, respectively). In the middle-high SDI regions, the prevalence of NSNIs increased from 371.06/100,000 in 2019 to 378.61/100,000 in 2021. The burden of NSNIs in the Western Pacific region has been steadily rising, with incidence increasing from 16,489.15/100,000 in 2017 to 17,608.97/100,000 in 2021, prevalence rising from 214.96/100,000 in 2018 to 225.27/100,000 in 2021, DALYs from 53,165.45/100,000 in 2016 to 57,179.59/100,000 in 2021, and mortality rising from 586.92/100,000 in 2016 to 635.23/100,000 in 2021. In the Eastern Mediterranean, DALYs rose from 125,896.44/100,000 in 2018 to 131,698.77/100,000 in 2021, with mortality also increasing from 1,398.68/100,000 in 2018 to 1,463.22/100,000 in 2021 (Figure 1, Supplementary Figures S1–S3, Supplementary Tables S1–S4).

### 3.3 National trends of neonatal sepsis and other neonatal infections (NSNIs) from 1990 to 2021

In 1990, among 204 countries and regions, the three countries with the highest burden of DALYs were Mali (880,600.64/100,000), Guinea-Bissau (848,244.20/100,000), and Sierra Leone (846,073.26/100,000). Conversely, the three countries with the lowest DALYs burden were Taiwan (Province of China) (1,099.99/100,000), Albania (1,505.36/100,000), and Bulgaria (3,005.25/100,000). In terms of mortality burden in 1990, Mali, Guinea-Bissau, and Sierra Leone again reported the highest figures, with values of 9,786.05/100,000, 9,426.45/100,000, and 9,402.13/100,000, respectively. Conversely, the lowest mortality burdens were observed in Taiwan (Province of China) (11.70/100,000), Albania (14.86/100,000), and Bulgaria (31.83/100,000).

**Figure 2:**
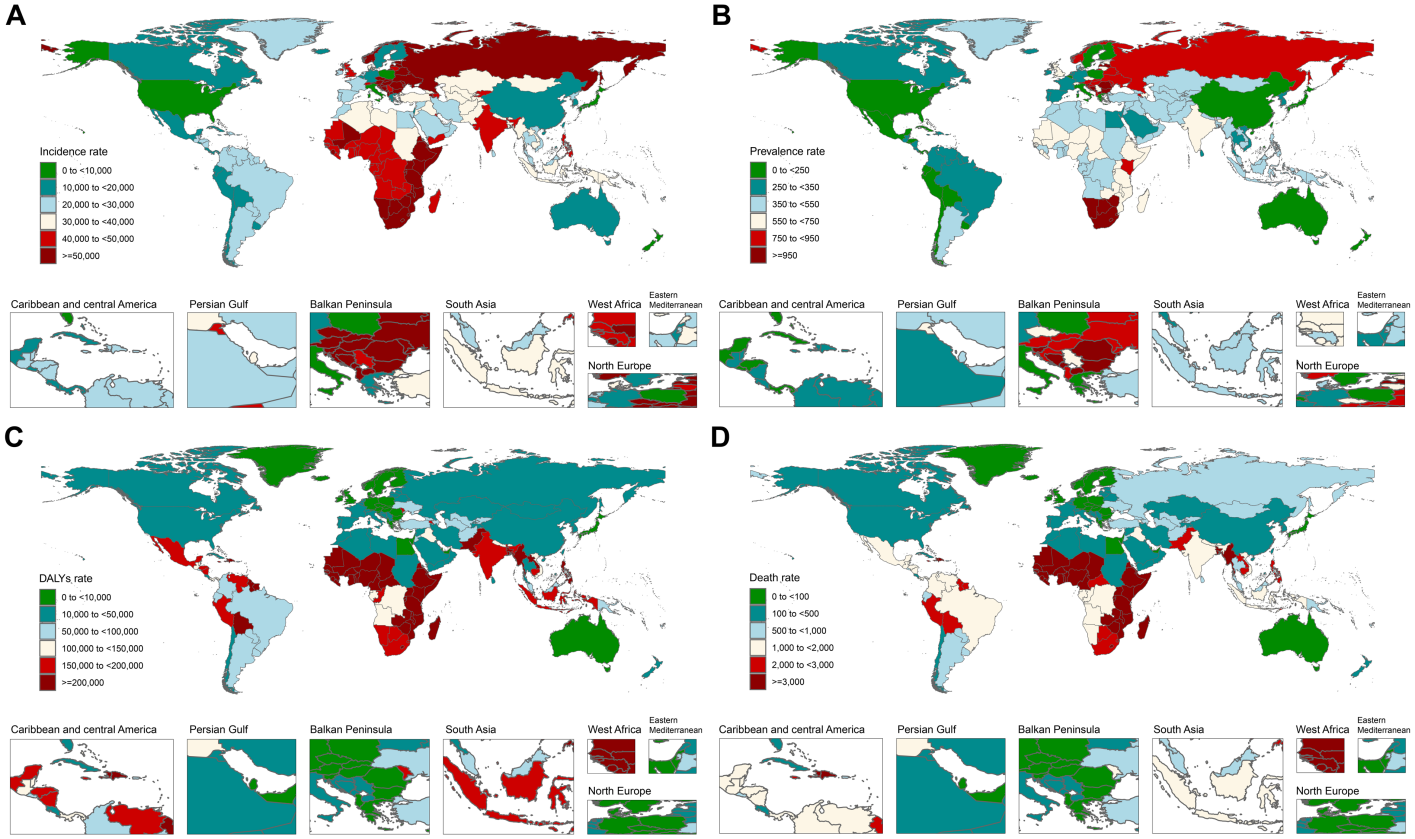
Distribution of NSNIs burden across 204 countries worldwide in 2021. **(A)** Incidence rate; **(B)** prevalence rate; **(C)** DALYs rate; and **(D)** death rate.

By 2021, the countries with the highest DALYs burden had shifted to Sierra Leone (534,090.25/100,000), Chad (520,317.08/100,000), and Burkina Faso (505,365.73/100,000), while the countries with the lowest DALYs were Norway (2,372.83/100,000), Singapore (2,547.13/100,000), and Qatar (2,597.42/100,000). In terms of mortality burden in 2021, Sierra Leone, Chad, and Burkina Faso continued to report the highest rates at 5,935.22/100,000, 5,782.14/100,000, and 5,616.07/100,000, respectively. On the other hand, the countries with the lowest mortality burdens were Norway (25.56/100,000), Singapore (28.11/100,000), and Qatar (28.27/100,000) (see Figure 2, Supplementary Figure S4).

### 3.4 Analysis of risk factors for neonatal sepsis and other neonatal infections (NSNIs) from 1990 to 2021

**Figure 3:**
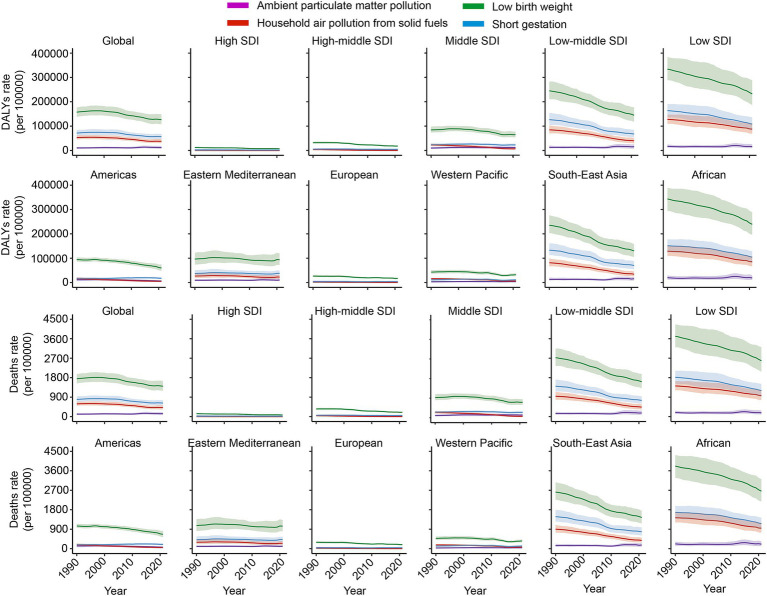
Trends in DALYs and mortality rates of NSNIs attributable to risk factors from 1990 to 2021. DALYs: disability-adjusted life years.

Globally, the DALYs associated with NSNIs due to ambient particulate matter pollution increased at an average annual rate of 0.7% from 1990 to 2021, rising from 10,372.41/100,000 in 1990 to 12,079.39/100,000 in 2021. Conversely, DALYs attributed to household air pollution from solid fuels decreased by 1.4%, declining from 52,915.19/100,000 in 1990 to 37,802.55/100,000 in 2021. The average annual change in DALYs due to low birth weight decreased by 0.9% (from 157,428.00/100,000 in 1990 to 126,333.02/100,000 in 2021), while DALYs attributed to short gestation declined by 1.0% (from 71,521.59/100,000 in 1990 to 56,455.75/100,000 in 2021) (Figure 3; Table 2).

**Table 2:** Trends in neonatal sepsis and other neonatal infections’ DALYs caused by risk factors globally and regionally from 1990 to 2021 (per 100,000).

Regions	Ambient particulate matter pollution			Household air pollution from solid fuels			Low birth weight			Short gestation		
	1990	2021	AAPC	1990	2021	AAPC	1990	2021	AAPC	1990	2021	AAPC
Global	10372.405 (6722.909, 14790.4)	12079.386 (7051.511, 18514.364)	0.7	52915.194 (45394.789, 61783.293)	37802.546 (29898.033, 47248.564)	−1.4	157428.002 (137868.082, 176973.519)	126333.016 (107609.749, 148647.296)	−0.9	71521.591 (60741.165, 84166.744)	56455.749 (45741.196, 67877.307)	−1.0
High SDI	2257.893 (1695.401, 2899.516)	728.697 (576.07, 916.909)	−3.9	218.567 (71.265, 533.919)	1.234 (0.002, 9.867)	−16.9	12146.453 (10739.059, 13796.735)	6862.911 (6064.517, 7682.739)	−2.0	1499.913 (1110.961, 1915.152)	1607.379 (1198.965, 1972.834)	−0.1
High-middle SDI	5136.441 (3257.679, 6829.672)	3003.407 (2275.756, 3792.417)	−2.1	3745.333 (2380.813, 5676.856)	97.919 (10.463, 552.519)	−12.2	31800.244 (27569.017, 36529.745)	18023.59 (15240.082, 21167.322)	−2.2	4715.219 (3254.464, 6399.508)	4610.279 (3452.575, 5801.922)	−0.6
Middle SDI	9239.693 (5240.319, 13717.343)	11388.807 (6833.751, 15633.763)	0.5	22090.38 (17227.811, 27780.65)	6128.814 (3230.577, 10730.41)	−4.5	83429.859 (72522.681, 97216.703)	63911.949 (53309.507, 76639.895)	−1.1	23254.334 (18741.362, 28181.359)	22282.383 (17120.345, 27666.894)	−0.3
Low-middle SDI	12975.012 (8039.541, 20277.43)	15246.186 (7845.518, 25187.833)	0.8	84716.852 (70016.876, 102769.582)	39488.557 (28965.927, 51270.451)	−2.6	245039.993 (210397.868, 282702.374)	144740.034 (117741.278, 177268.922)	−1.8	126537.048 (104429.75, 154103.016)	67382.972 (53887.255, 84432.679)	−2.2
Low SDI	17046.753 (11313.434, 24677.426)	15548.724 (9266.861, 25371.117)	0.5	127561.59 (109365.889, 147718.725)	87624.741 (69206.665, 109373.382)	−1.2	334074.055 (288514.729, 383586.15)	232398.795 (186803.415, 288662.761)	−1.1	163551.815 (136259.065, 190850.061)	108087.545 (81819.356, 135685.161)	−1.3
Americas	11439.653 (6472.583, 16838.967)	6274.18 (3820.526, 8588.524)	−1.9	16507.258 (11725.513, 22034.347)	4824.363 (2927.618, 7577.149)	−4.1	94181.212 (86442.073, 103478.858)	59707.787 (47526.044, 73960.113)	−1.4	16834.072 (13208.338, 20731.772)	17621.603 (13383.142, 22735.98)	0.5
European	4239.046 (2444.928, 6008.348)	2408.436 (1662.216, 3172.014)	−2.0	1555.561 (582.178, 3229.219)	445.496 (193.504, 1005.967)	−5.0	26412.577 (22098.524, 31680.794)	16893.995 (14536.678, 19762.823)	−1.5	3222.722 (1679.129, 5313.68)	3954.021 (2976.332, 5018.882)	0.3
Eastern Mediterranean	9422.744 (5917.213, 13977.516)	10394.084 (6319.492, 17256.18)	0.3	26666.942 (18460.757, 38113.198)	22695.329 (15640.148, 31599.639)	−1.0	96287.779 (73665.172, 122357.765)	93752.244 (72350.79, 119345.214)	−0.4	37265.506 (25989.031, 50858.076)	37913.64 (27325.235, 49675.471)	−0.4
Western Pacific	2944.387 (1754.199, 4602.763)	3827.157 (1965.062, 5916.212)	0.5	15560.024 (12370.513, 19652.309)	6849.402 (4789.943, 9321.721)	−3.3	42810.72 (36033.832, 50632.598)	32280.547 (26661.31, 38653.598)	−1.4	10927.693 (8107.172, 14385.828)	11284.15 (8209.78, 14511.862)	−0.5
South-East Asia	12861.595 (7817.271, 20655.947)	15338.75 (8553.843, 23515.838)	0.6	81229.84 (66428.258, 99360.104)	34301.904 (24958.29, 46017.698)	−2.9	235416.478 (203200.641, 276257.415)	129788.46 (104872.361, 157860.858)	−2.0	133160.842 (112797.712, 160979.427)	70098.173 (54276.099, 88160.598)	−2.3
African	19925.591 (12100.61, 29317.699)	18917.054 (10297.425, 30529.213)	0.7	128782.886 (109014.616, 151169.873)	85517.654 (66909.692, 107072.226)	−1.4	343512.05 (293993.805, 389469.756)	239168.254 (196194.843, 288606.199)	−1.1	151144.168 (123847.689, 177572.997)	104280.454 (80044.592, 129070.685)	−1.1

AAPC, average annual percentage change; DALY, disability-adjusted life year; SDI, sociodemographic index; UI, uncertainty interval.

From 1990 to 2021, DALYs related to NSNIs caused by ambient particulate matter pollution decreased by 3.9, 2.1, 1.9, and 2.0% in regions with high SDI, middle-high SDI, Americas, and Europe, respectively. In regions with high and middle-high SDI, DALYs resulting from household air pollution in solid fuels saw significant reductions of 16.9 and 12.2%, respectively. However, in the Americas and Europe, DALYs related to short gestation increased by 0.5 and 0.3%, respectively (Figure 3; Table 2).

It is noteworthy that as of now, DALYs resulting from household air pollution in solid fuels, low birth weight, and short gestation remain high in Africa and Southeast Asia. In 2021, the figures were 85,517.65/100,000, 239,168.25/100,000, and 104,280.45/100,000 for Africa, and 34,301.90/100,000, 129,788.46/100,000, and 70,098.17/100,000 for Southeast Asia, respectively, representing burdens far exceeding those in other regions. Recently, household air pollution from solid fuels has increased in the Eastern Mediterranean and Western Pacific regions, rising from 21,050.81/100,000 in 2018 to 22,695.33/100,000 in 2021 in the Eastern Mediterranean and from 6,100.55/100,000 in 2018 to 6,849.40/100,000 in the Western Pacific. Additionally, DALYs attributed to low birth weight have risen in both the Eastern Mediterranean and Western Pacific regions (from 88,653.41/100,000 in 2018 to 93,752.24/100,000 in the Eastern Mediterranean and from 28,813.84/100,000 in 2017 to 32,280.55/100,000 in the Western Pacific). Furthermore, short gestation has shown upward trends in regions with middle-high SDI, middle SDI, as well as in the Western Pacific and Eastern Mediterranean, increasing from 4,448.27/100,000 in 2016 to 4,610.28/100,000, from 21,283.08/100,000 in 2016 to 22,282.38/100,000, from 34,835.64/100,000 in 2017 to 37,913.64/100,000, and from 9,074.92/100,000 in 2016 to 11,284.15/100,000 (Figure 3; Table 2).

From 1990 to 2021, the mortality rate of NSNIs attributed to ambient particulate matter pollution increased by an average annual rate of 0.7%, rising from 115.24/100,000 in 1990 to 134.22/100,000 in 2021. In contrast, the mortality rate from household air pollution in solid fuels declined by 1.4% (from 587.97/100,000 in 1990 to 420.07/100,000 in 2021). The mortality rate associated with low birth weight decreased by 0.9% (from 1,749.70/100,000 in 1990 to 1,404.10/100,000 in 2021), while the mortality rate resulting from short gestation showed an annual change rate of −1.0%, remaining steady at 794.91/100,000 (Figure 3; Table 3).

**Table 3:** Trends in neonatal sepsis and other neonatal infections’ death caused by risk factors globally and regionally from 1990 to 2021 (per 100,000).

Regions	Ambient particulate matter pollution			Household air pollution from solid fuels			Low birth weight			Short gestation		
	1990	2021	AAPC	1990	2021	AAPC	1990	2021	AAPC	1990	2021	AAPC
Global	115.241 (74.688,164.311)	134.216 (78.345, 205.718)	0.7	587.965 (504.372, 686.499)	420.069 (332.233, 525.062)	−1.4	1749.695 (1532.301, 1966.926)	1404.095 (1, 196, 1652.101)	−0.9	794.909 (675.092, 935.45)	627.462 (508.378, 754.404)	−1.0
High SDI	25.069 (18.82, 32.2)	8.086 (6.392, 10.18)	−3.9	2.427 (0.791, 5.928)	0.014 (0.000, 0.109)	−16.9	134.999 (119.357, 153.341)	76.276 (67.403, 85.388)	−2.0	16.67 (12.348, 21.286)	17.865 (13.326, 21.927)	−0.1
High-middle SDI	57.036 (36.177, 75.839)	33.350 (25.266, 42.117)	−2.1	41.582 (26.435, 63.037)	1.087 (0.116, 6.134)	−12.2	353.436 (306.41, 406.001)	200.319 (169.382, 235.259)	−2.2	52.406 (36.171, 71.126)	51.24 (38.373, 64.484)	−0.6
Middle SDI	102.652 (58.214, 152.396)	126.533 (75.923, 173.706)	0.5	245.435 (191.398, 308.66)	68.099 (35.895, 119.233)	−4.5	927.261 (806.036, 1080.491)	710.333 (592.495, 851.794)	−1.1	258.455 (208.297, 313.215)	247.652 (190.28, 307.497)	−0.3
Low-middle SDI	144.166 (89.335, 225.326)	169.403 (87.162, 279.896)	0.8	941.334 (778.045, 1141.953)	438.792 (321.872, 569.699)	−2.6	2723.435 (2338.414, 3142.022)	1608.675 (1308.605, 1970.208)	−1.8	1406.363 (1160.658, 1712.735)	748.91 (598.916, 938.404)	−2.2
Low SDI	189.424 (125.719, 274.205)	172.784 (102.974, 281.941)	0.5	1417.457 (1215.266, 1641.565)	973.721 (769.019, 1215.448)	−1.2	3712.985 (3206.628, 4263.266)	2582.934 (2076.177, 3208.265)	−1.1	1817.755 (1514.419, 2121.151)	1201.31 (909.36, 1508.034)	-1.3
Americas	127.112 (71.918, 187.11)	69.718 (42.452, 95.438)	−1.9	183.437 (130.301, 244.856)	53.612 (32.532, 84.206)	−4.1	1046.756 (960.741, 1150.092)	663.608 (528.217, 822.012)	−1.4	187.099 (146.802, 230.419)	195.851 (148.744, 252.694)	0.5
European	47.051 (27.132, 66.649)	26.738 (18.449, 35.225)	−1.9	17.267 (6.466, 35.857)	4.947 (2.148, 11.171)	−5.0	293.557 (245.609, 352.109)	187.764 (161.565, 219.649)	−1.5	35.818 (18.662, 59.058)	43.946 (33.08, 55.781)	0.3
Eastern Mediterranean	104.654 (65.725, 155.197)	115.47 (70.199, 191.743)	0.3	296.270 (205.065, 423.468)	252.183 (173.79, 351.131)	−1.0	1070.168 (818.734, 1359.916)	1041.986 (804.126, 1326.431)	−0.4	414.178 (288.848, 565.249)	421.382 (303.7, 552.105)	−0.4
Western Pacific	32.707 (19.489, 51.14)	42.515 (21.827, 65.728)	0.5	172.867 (137.439, 218.31)	76.104 (53.221, 103.579)	−3.3	475.809 (400.489, 562.743)	358.774 (296.32, 429.605)	−1.4	121.453 (90.105, 159.888)	125.415 (91.246, 161.288)	−0.5
South-East Asia	142.905 (86.849, 229.495)	170.426 (95.037, 261.278)	0.6	902.567 (738.002, 1104.011)	381.147 (277.325, 511.33)	−2.9	2616.475 (2258.42, 3070.387)	1442.499 (1165.576, 1754.502)	−2.0	1479.982 (1253.662, 1789.163)	779.088 (603.238, 979.837)	−2.3
African	221.414 (134.469, 325.761)	210.214 (114.43, 339.255)	0.7	1431.051 (1211.412, 1679.823)	950.316 (743.49, 1189.902)	−1.4	3817.882 (3267.523, 4328.666)	2658.171 (2180.555, 3207.636)	−1.1	1679.854 (1376.476, 1973.587)	1158.997 (889.634, 1434.521)	−1.1

AAPC, average annual percentage change; SDI, sociodemographic index; UI, uncertainty interval.

During the period from 1990 to 2021, in regions with high SDI, middle-high SDI, as well as in the Americas and Europe, the mortality rates caused by ambient particulate matter pollution decreased by 3.9, 2.1, 1.9, and 1.9%, respectively. In high and middle-high SDI regions, the mortality rates related to household air pollution from solid fuels also significantly decreased by 16.9 and 12.2%, respectively. In the Americas and Europe, however, mortality rates due to short gestation increased by 0.5 and 0.3%, respectively (Figure 3; Table 3).

It is crucial to emphasize that, as of now, the mortality rates from NSNIs attributed to household air pollution from solid fuels, low birth weight, and short gestation remain alarmingly high in Africa and Southeast Asia. In 2021, the figures were 950.31/100,000, 2,658.17/100,000, and 1,159.00/100,000 for Africa, and 381.15/100,000, 1,442.50/100,000, and 779.09/100,000 for Southeast Asia, respectively. These burdens far exceed those in other regions. Moreover, there has been a recent increase in household air pollution from solid fuels in the Eastern Mediterranean and Western Pacific regions, with rates rising from 233.90/100,000 in 2018 to 252.18/100,000 in 2021 in the Eastern Mediterranean and from 67.78/100,000 in 2018 to 76.10/100,000 in the Western Pacific. Additionally, the mortality rates associated with low birth weight have risen in the Western Pacific and Eastern Mediterranean (from 985.31/100,000 in 2018 to 1,041.99/100,000 in the Eastern Mediterranean and from 320.24/100,000 in 2017 to 358.77/100,000 in the Western Pacific). Furthermore, short gestation has exhibited increasing trends in regions with middle-high SDI, middle SDI, Western Pacific, and Eastern Mediterranean, increasing from 49.44/100,000 in 2016 to 51.24/100,000, from 236.55/100,000 in 2016 to 247.65/100,000, from 387.17/100,000 in 2017 to 421.38/100,000, and from 100.86/100,000 in 2016 to 125.41/100,000 (Figure 3; Table 3).

## 4 Discussion

This study analyzed the global, regional, and national trends of NSNIs from 1990 to 2021. The results indicate a significant decline in the incidence, prevalence, DALYs, and mortality associated with NSNIs worldwide, reflecting improvements in healthcare and sanitary conditions. This demonstrates the effectiveness of public health interventions and neonatal care practices (26). However, the burden of neonatal sepsis and other infections remains high in the African and Southeast Asian regions, primarily influenced by the following factors:

Firstly, insufficient medical resources present a key challenge, including a shortage of healthcare facilities, a lack of skilled healthcare personnel, and low vaccination coverage (27–29). These factors hinder timely interventions and care for newborns, leading to the recurrence of preventable diseases. Additionally, poor environmental sanitation, a lack of safe drinking water, and inadequate basic hygiene facilities, compounded by natural disasters and climate change, place extra pressure on public health systems and further exacerbate the risks to neonatal health (30). Socioeconomic factors also play a significant role (31). Poverty and low levels of economic development mean that many families lack sufficient nutrition and sanitary conditions, increasing the risk of neonatal infections. Furthermore, maternal and infant malnutrition weakens the immune system, making newborns more susceptible to infections. Cultural factors and a lack of public knowledge regarding neonatal care further hinder the implementation of effective health protection measures. The rising issue of antibiotic resistance, driven by misuse and overuse of antibiotics, complicates and challenges the treatment of common infections (32, 33). Regional conflicts and wars result in social unrest that further affects maternal and child health and the accessibility of medical services (34, 35).

In the Eastern Mediterranean region, persistent public health crises, conflicts, declining vaccination rates, and escalating environmental health issues have led to a significant increase in the burden of neonatal sepsis and other infections (36). Similarly, the Western Pacific region faces challenges such as weak public health infrastructure, insufficient vaccination coverage, climate change, and socioeconomic factors that collectively heighten the risk of neonatal infections (37, 38).

Despite the higher burden of NSNIs in Africa and Southeast Asia, some countries have made significant progress in improving neonatal health. The proactive implementation of vaccination programs and maternal and child health initiatives has significantly reduced the incidence of neonatal infections. This indicates that policy-driven interventions can indeed improve neonatal health outcomes, providing valuable lessons for other high-burden regions (39–41). According to WHO reports, improving environmental sanitation, water quality and its availability, alongside appropriate hand hygiene and infection control measures, can prevent sepsis and save lives. However, these measures must be complemented by early diagnosis, appropriate clinical management, and access to safe and affordable medications and vaccines. Such interventions could prevent up to 84% of neonatal deaths due to sepsis (42). This emphasizes the effectiveness of targeted measures in reducing the burden of neonatal infections, offering guidance for policymakers in Africa and Southeast Asia.

In summary, the increasing burden of neonatal infections in Africa, Southeast Asia, the Eastern Mediterranean, and the Western Pacific regions is multifaceted. Addressing these issues urgently requires systematic interventions to improve public health conditions, enhance vaccination coverage, and strengthen maternal and child health services to ensure the well-being of newborns. These efforts are crucial to reducing the infection burden in these regions. Furthermore, this study has some limitations. First, the availability and completeness of data may introduce potential reporting biases. In regions with inadequate healthcare systems or ideological influences, neonatal sepsis and other infections may not be accurately diagnosed or reported. This bias could result in discrepancies between the actual incidence and mortality rates and the data presented in this study. Second, certain potential influencing factors were not adequately considered. Although various environmental and socioeconomic factors impacting the burden of NSNIs were analyzed, other potential factors such as health policy, cultural influences, and healthcare infrastructure may exert differing effects on disease burden in different regions. Third, the ecological study design employed in this research has inherent limitations that restrict our ability to establish causal relationships. While observed trends may suggest potential associations, the causal relationships among the related variables have not been comprehensively identified.

## 5 Conclusion

This study reveals the changing trends in the global burden of NSNIs from 1990 to 2021. While an overall downward trend is observed globally, the burden of NSNIs remains substantial in Africa and Southeast Asia. Alarmingly, we identified an increasing trend in the Eastern Mediterranean and Western Pacific regions in recent years, which calls for targeted public health interventions. Additionally, our findings indicate that neonatal infections are notably associated with risk factors such as short gestation, low birth weight, and environmental factors like particulate pollution. Addressing these risk factors through comprehensive intervention programs is essential for improving neonatal survival rates. Furthermore, the management of antibiotic use must be strengthened within the global health community to combat neonatal sepsis and other infections, particularly in light of the growing concern over antibiotic resistance. It is imperative to implement evidence-based policies that focus on prevention strategies tailored to high-burden areas. In summary, this study underscores the importance of continued vigilance and adaptive public health strategies to address the persistent challenges posed by neonatal infections, ensuring that the progress made in some regions does not overshadow the ongoing plight in others. Future research should further explore the impact of socio-economic factors and healthcare access on neonatal infection rates to inform more effective intervention strategies.

## Data Availability

The raw data supporting the conclusions of this article will be made available by the authors, without undue reservation.

## Supplementary material

SUPPLEMENTARY FIGURE S1 Trends of prevalence rate of NSNIs at the global and regions from 1990 to 2021.

SUPPLEMENTARY FIGURE S2 Trends of DALYs rate of NSNIs at the global and regions from 1990 to 2021.

SUPPLEMENTARY FIGURE S3 Trends of death rate of NSNIs at the global and regions from 1990 to 2021.

SUPPLEMENTARY FIGURE S4 Distribution of NSNIs burden across 204 countries worldwide in 1990.
