# Supplementary figures and images for: Analysis of the global burden and key risk factors of neonatal sepsis and other neonatal infections in 204 countries and territories, 1990–2021
Source: Front Med (Lausanne). 2025 Apr 4;12:1536948. doi: 10.3389/fmed.2025.1536948 (PMC12006091; doi:10.3389/fmed.2025.1536948)

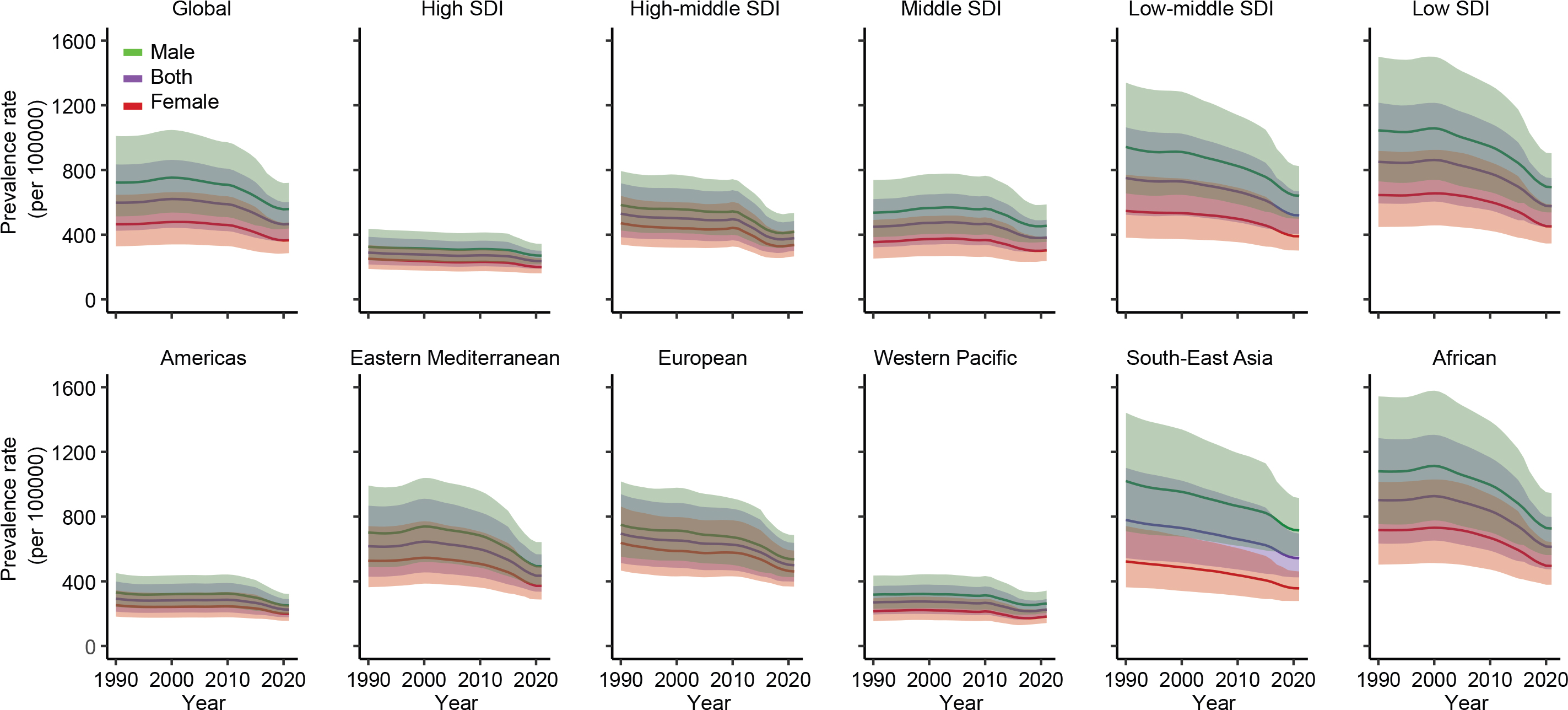

Supplement: SUPPLEMENTARY FIGURE S1 — Trends of prevalence rate of NSNIs at the global and regions from 1990 to 2021. [file Image_1.jpeg]

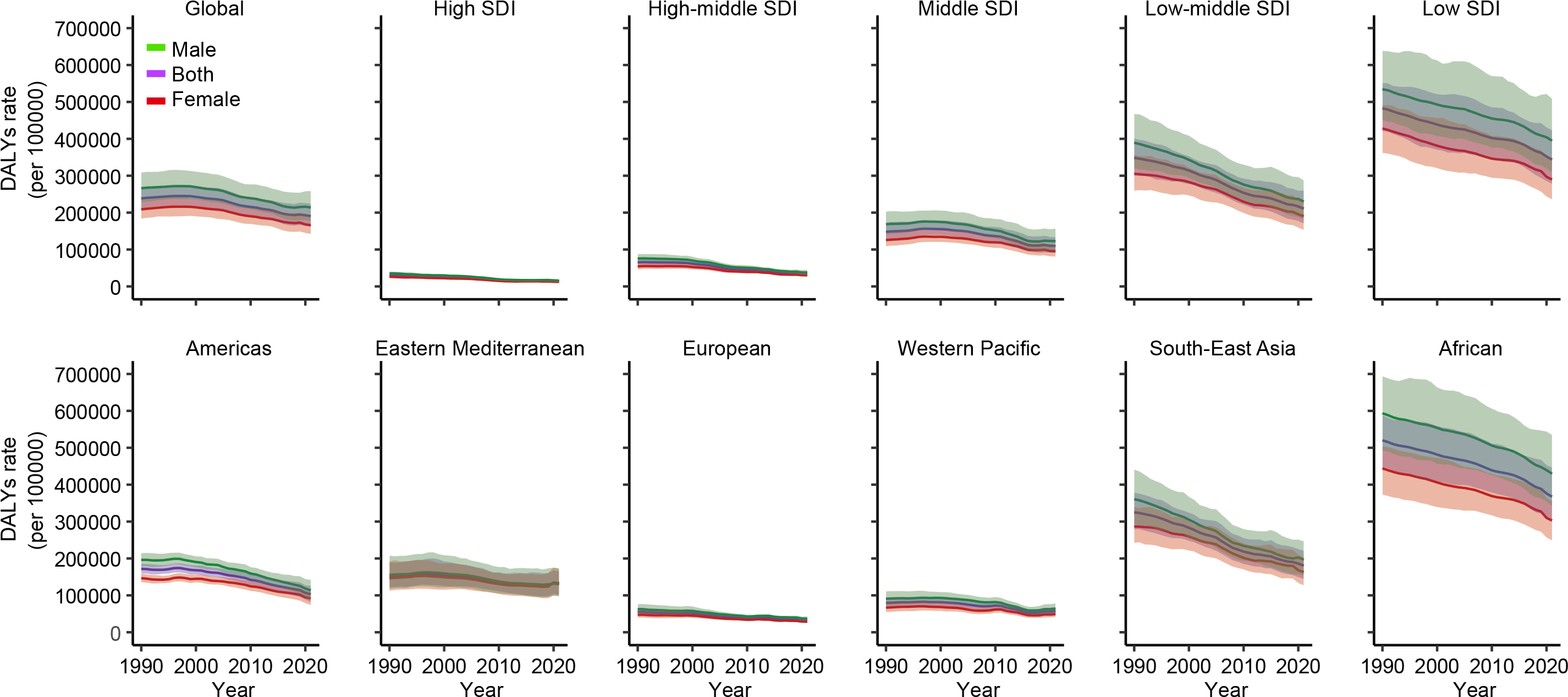

Supplement: SUPPLEMENTARY FIGURE S2 — Trends of DALYs rate of NSNIs at the global and regions from 1990 to 2021. [file Image_2.jpeg]

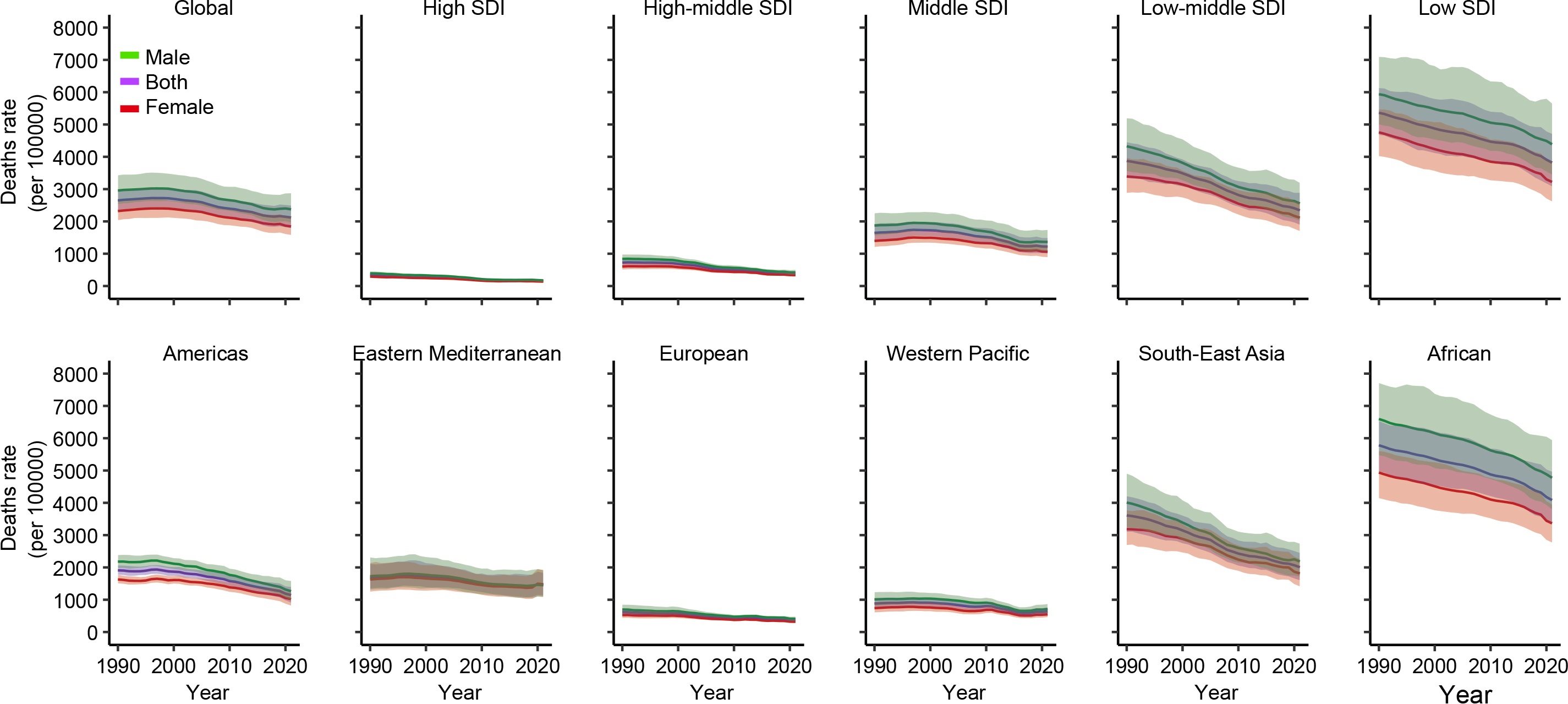

Supplement: SUPPLEMENTARY FIGURE S3 — Trends of death rate of NSNIs at the global and regions from 1990 to 2021. [file Image_3.jpeg]

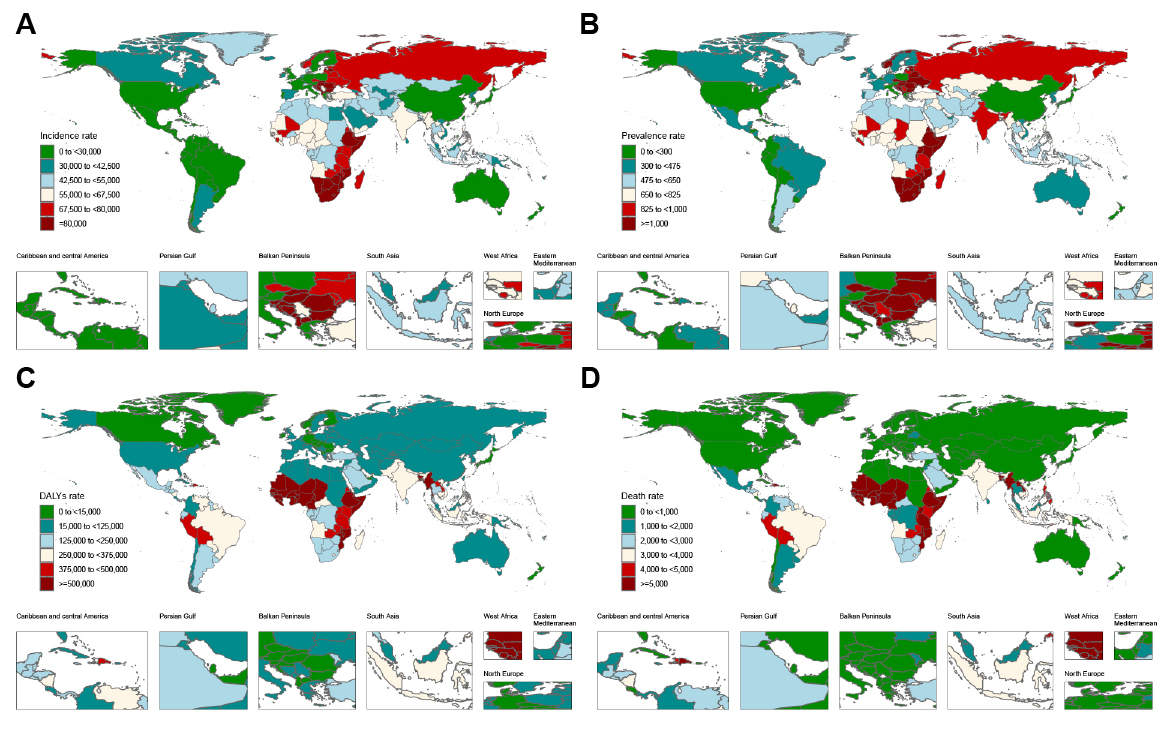

Supplement: SUPPLEMENTARY FIGURE S4 — Distribution of NSNIs burden across 204 countries worldwide in 1990. [file Image_4.jpeg]
