# Supplementary material for: Analysis of the global burden and key risk factors of neonatal sepsis and other neonatal infections in 204 countries and territories, 1990–2021
Source: Front Med (Lausanne). 2025 Apr 4;12:1536948. doi: 10.3389/fmed.2025.1536948 (PMC12006091; doi:10.3389/fmed.2025.1536948)
Supplement: Supplementary file 5 [file Table_1.docx]

| **Table S1. Trends in the incidence of neonatal sepsis and other neonatal infections globally and regionally from 1990 to 2021** | | |
| --- | --- | --- |
| Region | Year | Incidence rate(95%UI) |
| African | 1990 | 71292.173(69237.458,73475.626) |
|  | 1991 | 71230.515(69217.239,73424.65) |
|  | 1992 | 71209.355(69196.808,73428.348) |
|  | 1993 | 71210.049(69177.733,73426.223) |
|  | 1994 | 71233.928(69161.807,73453.129) |
|  | 1995 | 71319.574(69183.113,73570.17) |
|  | 1996 | 71556.696(69358.441,73800.959) |
|  | 1997 | 71941.758(69781.493,74173.92) |
|  | 1998 | 72334.904(70259.233,74569.685) |
|  | 1999 | 72621.582(70522.237,74941.849) |
|  | 2000 | 72687.094(70444.33,75096.795) |
|  | 2001 | 72434.418(70278.579,74791.186) |
|  | 2002 | 71908.065(69727.185,74197.454) |
|  | 2003 | 71196.854(69109.426,73425.894) |
|  | 2004 | 70400.361(68299.023,72551.863) |
|  | 2005 | 69612.195(67533.439,71754.013) |
|  | 2006 | 68822.509(66841.347,70866.872) |
|  | 2007 | 67971.985(66032.67,69922.892) |
|  | 2008 | 67065.281(65045.05,68942.564) |
|  | 2009 | 66103.601(64143.38,67894.982) |
|  | 2010 | 65088.99(63143.014,66913.413) |
|  | 2011 | 63839.451(61981.089,65562.062) |
|  | 2012 | 62281.816(60450.473,63933.964) |
|  | 2013 | 60577.579(58804.838,62168.465) |
|  | 2014 | 58883.204(57170.398,60430.001) |
|  | 2015 | 57352.015(55651.063,58862.657) |
|  | 2016 | 56031.472(54358.377,57457.117) |
|  | 2017 | 54791.309(53146.483,56188.464) |
|  | 2018 | 53543.199(51938.978,54908.96) |
|  | 2019 | 52189.229(50665.762,53605.438) |
|  | 2020 | 50578.512(49155.432,51973.889) |
|  | 2021 | 50143.032(48733.947,51524.93) |
| Americas | 1990 | 22281.248(21900.623,22683.099) |
|  | 1991 | 21945.256(21589.235,22344.027) |
|  | 1992 | 21669.213(21322.281,22059.775) |
|  | 1993 | 21454.869(21097.119,21832.253) |
|  | 1994 | 21299.053(20945.207,21676.632) |
|  | 1995 | 21219.63(20850.133,21604.79) |
|  | 1996 | 21199.932(20838.553,21560.839) |
|  | 1997 | 21206.401(20838.954,21553.053) |
|  | 1998 | 21222.724(20861.841,21557.669) |
|  | 1999 | 21226.475(20873.45,21554.236) |
|  | 2000 | 21229.771(20873.37,21568.834) |
|  | 2001 | 21248.345(20903.046,21592.527) |
|  | 2002 | 21264.537(20928.165,21606.008) |
|  | 2003 | 21275.174(20935.776,21622.605) |
|  | 2004 | 21273.59(20935.56,21633.958) |
|  | 2005 | 21226.772(20883.874,21591.991) |
|  | 2006 | 21182.132(20857.577,21531.615) |
|  | 2007 | 21209.363(20891.743,21550.811) |
|  | 2008 | 21282.369(20967.062,21620.405) |
|  | 2009 | 21346.105(21016.923,21687.192) |
|  | 2010 | 21351.461(21020.54,21701.346) |
|  | 2011 | 21210.985(20888.235,21552.369) |
|  | 2012 | 20932.191(20625.859,21269.91) |
|  | 2013 | 20573.741(20283.997,20902.209) |
|  | 2014 | 20195.421(19905.74,20530.491) |
|  | 2015 | 19863.483(19573.023,20212.897) |
|  | 2016 | 19518.22(19243.471,19856.276) |
|  | 2017 | 19085.423(18801.82,19412.455) |
|  | 2018 | 18588.253(18306.636,18899.533) |
|  | 2019 | 18120.933(17838.704,18425.779) |
|  | 2020 | 17679.49(17406.969,17978.514) |
|  | 2021 | 17512.135(17236.945,17803.335) |
| Eastern Mediterranean | 1990 | 47863.675(45881.804,50140.446) |
|  | 1991 | 47746.388(45731.471,49914.664) |
|  | 1992 | 47661.12(45689.593,49760.911) |
|  | 1993 | 47654.447(45653.89,49809.578) |
|  | 1994 | 47769.058(45677.307,49951.548) |
|  | 1995 | 47938.529(45738.078,50178.454) |
|  | 1996 | 48229.424(46033.708,50419.287) |
|  | 1997 | 48682.455(46495.722,50864.59) |
|  | 1998 | 49173.791(46947.718,51376.503) |
|  | 1999 | 49589.228(47402.481,51841.602) |
|  | 2000 | 49809.363(47642.255,52139.07) |
|  | 2001 | 49768.664(47690.035,51973.997) |
|  | 2002 | 49510.393(47385.358,51692.118) |
|  | 2003 | 49114.969(46943.182,51292.481) |
|  | 2004 | 48698.525(46586.345,50769.127) |
|  | 2005 | 48339.355(46135.639,50444.266) |
|  | 2006 | 47966.226(45860.007,50035.167) |
|  | 2007 | 47486.503(45318.62,49512.844) |
|  | 2008 | 46936.347(44811.042,48973.482) |
|  | 2009 | 46319.665(44160.747,48385.205) |
|  | 2010 | 45635.567(43468.067,47736.728) |
|  | 2011 | 44784.674(42713.943,46854.957) |
|  | 2012 | 43748.721(41695.162,45760.982) |
|  | 2013 | 42645.811(40624.221,44564.736) |
|  | 2014 | 41530.15(39556.337,43436.697) |
|  | 2015 | 40467.786(38536.836,42395.654) |
|  | 2016 | 39471.208(37608,41398.714) |
|  | 2017 | 38470.547(36633.864,40393.778) |
|  | 2018 | 37441.921(35610.196,39348.344) |
|  | 2019 | 36370.69(34546.177,38307.048) |
|  | 2020 | 35185.394(33407.128,37111.898) |
|  | 2021 | 34965.464(33253.029,36881.244) |
| European | 1990 | 51725.193(49952.525,53562.293) |
|  | 1991 | 50944.359(49326.658,52694.054) |
|  | 1992 | 50304.455(48737.294,51991.683) |
|  | 1993 | 49844.46(48310.601,51508.79) |
|  | 1994 | 49475.468(48000.248,51132.592) |
|  | 1995 | 49058.735(47658.142,50709.267) |
|  | 1996 | 48657.75(47260.124,50246.517) |
|  | 1997 | 48418.813(47021.731,49903.185) |
|  | 1998 | 48276.176(46896.714,49722.021) |
|  | 1999 | 48135.81(46735.378,49550.813) |
|  | 2000 | 48043.491(46547.776,49532.061) |
|  | 2001 | 47900.687(46435.47,49389.829) |
|  | 2002 | 47593.923(46123.919,49090.415) |
|  | 2003 | 47099.441(45653.817,48637.571) |
|  | 2004 | 46591.381(45155.422,48065.645) |
|  | 2005 | 46292.141(44780.58,47775.285) |
|  | 2006 | 46173.949(44712.259,47676.805) |
|  | 2007 | 46091.415(44607.179,47598.158) |
|  | 2008 | 46011.47(44507.89,47550.354) |
|  | 2009 | 45841.345(44297.753,47402.769) |
|  | 2010 | 45628.441(44026.551,47269.903) |
|  | 2011 | 45301.193(43756.506,46910.105) |
|  | 2012 | 44669.181(43136.915,46251.699) |
|  | 2013 | 43811.997(42342.413,45334.793) |
|  | 2014 | 42902.63(41463.039,44398.478) |
|  | 2015 | 42050.089(40637.121,43547.792) |
|  | 2016 | 41181.499(39865.324,42580.504) |
|  | 2017 | 40304.469(39049.572,41599.677) |
|  | 2018 | 39527.52(38306.532,40804.595) |
|  | 2019 | 38886.92(37678.296,40143.202) |
|  | 2020 | 38293.765(37129.151,39453.404) |
|  | 2021 | 38003.222(36853.03,39115.27) |
| Global | 1990 | 46365.63(45720.759,47080.005) |
|  | 1991 | 46359.82(45764.874,47064.141) |
|  | 1992 | 46405.327(45803.689,47122.985) |
|  | 1993 | 46465.89(45830.016,47187.29) |
|  | 1994 | 46562.053(45885.067,47274.223) |
|  | 1995 | 46729.892(46053.373,47450.88) |
|  | 1996 | 47003.132(46311.477,47733.509) |
|  | 1997 | 47328.003(46647.697,48042.218) |
|  | 1998 | 47601.195(46893.298,48312.154) |
|  | 1999 | 47800.039(47092.687,48516.809) |
|  | 2000 | 47865.432(47164.621,48581.879) |
|  | 2001 | 47797.28(47078.53,48508.101) |
|  | 2002 | 47679.456(46965.424,48402.48) |
|  | 2003 | 47472.754(46760.108,48201.497) |
|  | 2004 | 47152.522(46442.514,47901.29) |
|  | 2005 | 46778.497(46050.152,47537.094) |
|  | 2006 | 46372.847(45654.593,47093.872) |
|  | 2007 | 45932.472(45230.312,46642.693) |
|  | 2008 | 45535.819(44848.821,46238.042) |
|  | 2009 | 45203.27(44537.29,45908.201) |
|  | 2010 | 44998.772(44350.491,45687.35) |
|  | 2011 | 44419.475(43787.862,45084.551) |
|  | 2012 | 43362.138(42749.822,43984.259) |
|  | 2013 | 42347.278(41753.483,42967.939) |
|  | 2014 | 41316.034(40729.094,41933.374) |
|  | 2015 | 40168.24(39585.916,40775.401) |
|  | 2016 | 39233.31(38668.026,39836.234) |
|  | 2017 | 38670.954(38120.43,39266.791) |
|  | 2018 | 38212.479(37668.993,38811.855) |
|  | 2019 | 37827.061(37272.901,38430.802) |
|  | 2020 | 37355.412(36791.944,37963.77) |
|  | 2021 | 37294.434(36742.274,37915.165) |
| High SDI | 1990 | 20978.716(20550.698,21452.546) |
|  | 1991 | 20832.41(20403.233,21301.523) |
|  | 1992 | 20683.981(20264.638,21161.701) |
|  | 1993 | 20523.091(20128.285,20987.672) |
|  | 1994 | 20389.288(19995.055,20838.959) |
|  | 1995 | 20300.033(19906.154,20766.84) |
|  | 1996 | 20218.299(19839.106,20666.184) |
|  | 1997 | 20138.602(19751.984,20568.36) |
|  | 1998 | 20063(19698.776,20478.881) |
|  | 1999 | 19979.799(19613.095,20393.034) |
|  | 2000 | 19889.384(19515.724,20310.136) |
|  | 2001 | 19774.264(19407.167,20185.617) |
|  | 2002 | 19654.667(19298.051,20040.033) |
|  | 2003 | 19532.322(19192.024,19899.207) |
|  | 2004 | 19435.18(19109.716,19789.021) |
|  | 2005 | 19336.543(19005.526,19687.616) |
|  | 2006 | 19267.052(18951.934,19613.522) |
|  | 2007 | 19281.338(18975.774,19624.833) |
|  | 2008 | 19344.109(19043.37,19685.165) |
|  | 2009 | 19409.905(19104.25,19744.703) |
|  | 2010 | 19451.244(19132.293,19787.463) |
|  | 2011 | 19437.619(19127.089,19783.938) |
|  | 2012 | 19357.674(19057.731,19705.142) |
|  | 2013 | 19245.296(18939.062,19593.286) |
|  | 2014 | 19135.64(18832.926,19471.171) |
|  | 2015 | 18998.942(18687.879,19340.742) |
|  | 2016 | 18794.878(18484.475,19124.161) |
|  | 2017 | 18514.767(18212.975,18836.09) |
|  | 2018 | 18223.289(17924.721,18553.197) |
|  | 2019 | 17969.03(17668.797,18294.01) |
|  | 2020 | 17709.391(17408.693,18029.362) |
|  | 2021 | 17621.971(17315.654,17937.981) |
| High-middle SDI | 1990 | 39920.957(38599.642,41275.068) |
|  | 1991 | 39406.323(38168.244,40684.453) |
|  | 1992 | 38927.518(37772.169,40171.145) |
|  | 1993 | 38528.823(37410.67,39766.908) |
|  | 1994 | 38238.851(37115.452,39502.619) |
|  | 1995 | 37985.543(36848.068,39262.469) |
|  | 1996 | 37836.936(36729.121,39069.023) |
|  | 1997 | 37763.808(36674.367,39010.324) |
|  | 1998 | 37657.165(36545.08,38882.84) |
|  | 1999 | 37558.518(36428.086,38828.735) |
|  | 2000 | 37404.781(36215.945,38643.479) |
|  | 2001 | 37188.23(36017.612,38439.123) |
|  | 2002 | 37067.317(35877.778,38312.563) |
|  | 2003 | 36869.837(35696.051,38077.909) |
|  | 2004 | 36506.78(35362.256,37680.413) |
|  | 2005 | 36210.42(35018.049,37366.457) |
|  | 2006 | 36082.45(34939.457,37249.437) |
|  | 2007 | 36007.671(34838.923,37169.532) |
|  | 2008 | 35986.122(34799.057,37188.903) |
|  | 2009 | 36014.348(34844.506,37245.17) |
|  | 2010 | 36333.01(35121.684,37623.219) |
|  | 2011 | 35886.075(34705.777,37145.651) |
|  | 2012 | 34387.157(33244.918,35577.164) |
|  | 2013 | 33034.966(31934.887,34148.712) |
|  | 2014 | 31687.501(30625.114,32761.612) |
|  | 2015 | 30053.33(29045.704,31064.891) |
|  | 2016 | 28815.595(27901.163,29725.854) |
|  | 2017 | 28361.028(27465.061,29254.112) |
|  | 2018 | 28217.763(27319.975,29123.035) |
|  | 2019 | 28387.493(27485.795,29331.316) |
|  | 2020 | 28700.784(27739.629,29682.021) |
|  | 2021 | 28920.432(27952.855,29925.588) |
| Low SDI | 1990 | 67431.595(65432.735,69469.925) |
|  | 1991 | 67304.394(65317.229,69295.07) |
|  | 1992 | 67164.112(65223.572,69117.64) |
|  | 1993 | 66984.803(65183.913,68871.188) |
|  | 1994 | 66837.321(64988.477,68683.369) |
|  | 1995 | 66802.651(64914.001,68726.792) |
|  | 1996 | 66947.141(65057.84,68849.257) |
|  | 1997 | 67232.064(65258.422,69164.005) |
|  | 1998 | 67524.817(65540.857,69496.653) |
|  | 1999 | 67734.7(65729.369,69727.762) |
|  | 2000 | 67782.416(65723.811,69805.704) |
|  | 2001 | 67543.074(65482.579,69564.208) |
|  | 2002 | 66982.965(64969.943,68928.26) |
|  | 2003 | 66218.932(64263.275,68094.762) |
|  | 2004 | 65424.523(63529.39,67261.564) |
|  | 2005 | 64694.031(62826.248,66459.26) |
|  | 2006 | 63987.043(62217.273,65685.866) |
|  | 2007 | 63248.791(61490.009,64930.885) |
|  | 2008 | 62474.459(60797.235,64140.914) |
|  | 2009 | 61637.827(60013.76,63312.271) |
|  | 2010 | 60757.232(59165.152,62441.718) |
|  | 2011 | 59679.517(58180.364,61324.179) |
|  | 2012 | 58333.013(56910.577,59912.338) |
|  | 2013 | 56863.789(55464.007,58427.446) |
|  | 2014 | 55384.156(54031.739,56902.063) |
|  | 2015 | 54013.665(52697.159,55501.767) |
|  | 2016 | 52784.013(51529.772,54157.567) |
|  | 2017 | 51589.442(50366.447,52907.195) |
|  | 2018 | 50381.399(49138.084,51673.449) |
|  | 2019 | 49105.61(47847.006,50431.14) |
|  | 2020 | 47642.934(46442.983,48944.956) |
|  | 2021 | 47243.06(46033.556,48552.485) |
| Low-middle SDI | 1990 | 58459.896(56805.514,60132.325) |
|  | 1991 | 58042.599(56439.8,59652.404) |
|  | 1992 | 57684.187(56096.626,59275.604) |
|  | 1993 | 57378.973(55798.634,58983.408) |
|  | 1994 | 57122.608(55533.538,58693.44) |
|  | 1995 | 56924.698(55310.42,58503.231) |
|  | 1996 | 56800.528(55217.457,58298.536) |
|  | 1997 | 56753.04(55162.334,58222.295) |
|  | 1998 | 56723.877(55122.856,58131.377) |
|  | 1999 | 56646.868(55054.211,58031.505) |
|  | 2000 | 56466.888(54874.904,57896.197) |
|  | 2001 | 56149.085(54677.07,57550.147) |
|  | 2002 | 55724.741(54265.273,57132.85) |
|  | 2003 | 55222.778(53773.888,56606.174) |
|  | 2004 | 54703.093(53254.564,56075.262) |
|  | 2005 | 54225.956(52778.892,55627.651) |
|  | 2006 | 53742.707(52347.062,55124.21) |
|  | 2007 | 53191.255(51863.233,54573.323) |
|  | 2008 | 52584.7(51272.707,53939.867) |
|  | 2009 | 51933.755(50695.032,53298.694) |
|  | 2010 | 51258.26(49988.936,52619.592) |
|  | 2011 | 50484.615(49270.667,51801.014) |
|  | 2012 | 49577.94(48457.02,50814.658) |
|  | 2013 | 48615.344(47463.812,49820.39) |
|  | 2014 | 47647.557(46478.731,48857.746) |
|  | 2015 | 46736.605(45528.558,47945.304) |
|  | 2016 | 45865.45(44742.362,47029.926) |
|  | 2017 | 44975.108(43864.025,46152.056) |
|  | 2018 | 44081.429(42987.128,45250.327) |
|  | 2019 | 43188.735(42084.426,44372.328) |
|  | 2020 | 42222.214(41130.093,43350.34) |
|  | 2021 | 41836.527(40745.69,42942.435) |
| Middle SDI | 1990 | 34691.273(33915.05,35522.259) |
|  | 1991 | 34764.154(34003.96,35594.366) |
|  | 1992 | 34852.692(34105.401,35676.242) |
|  | 1993 | 34922.207(34190.23,35751.846) |
|  | 1994 | 35022.977(34272.24,35848.586) |
|  | 1995 | 35204.886(34457.799,36066.47) |
|  | 1996 | 35498.014(34784.043,36342.926) |
|  | 1997 | 35801.293(35110.114,36609.539) |
|  | 1998 | 36031.233(35320.671,36819.946) |
|  | 1999 | 36209.061(35482.508,37031.966) |
|  | 2000 | 36266.241(35519.794,37095.336) |
|  | 2001 | 36266.007(35535.92,37057.362) |
|  | 2002 | 36360.023(35652.83,37126.848) |
|  | 2003 | 36454.99(35746.917,37219.683) |
|  | 2004 | 36411.803(35664.298,37183.496) |
|  | 2005 | 36244.854(35472.92,37044.385) |
|  | 2006 | 35992.476(35232.076,36730.303) |
|  | 2007 | 35658.205(34921.687,36393.847) |
|  | 2008 | 35383.693(34670.426,36116.915) |
|  | 2009 | 35248.046(34526.576,36018.475) |
|  | 2010 | 35364.238(34631.413,36177.96) |
|  | 2011 | 35002.974(34287.818,35762.52) |
|  | 2012 | 34041.138(33326.604,34781.936) |
|  | 2013 | 33238.919(32511.932,33950.561) |
|  | 2014 | 32419.913(31697.874,33140.223) |
|  | 2015 | 31345.721(30641.343,32081.32) |
|  | 2016 | 30504.163(29821.744,31224.217) |
|  | 2017 | 30196.712(29503.765,30917.292) |
|  | 2018 | 30043.949(29344.202,30762.423) |
|  | 2019 | 30029.123(29331.706,30768.916) |
|  | 2020 | 30043.191(29331.853,30774.657) |
|  | 2021 | 30111.954(29367.125,30842.149) |
| South-East Asia | 1990 | 60712.485(58917.701,62436.694) |
|  | 1991 | 60151.585(58398.223,61813.877) |
|  | 1992 | 59627.803(57866.964,61218.795) |
|  | 1993 | 59136.395(57468.665,60723.057) |
|  | 1994 | 58684.768(57104.353,60323.406) |
|  | 1995 | 58289.197(56738.903,59982.433) |
|  | 1996 | 57922.704(56348.703,59567.702) |
|  | 1997 | 57564.684(55973.83,59197.085) |
|  | 1998 | 57196.899(55573.971,58826.731) |
|  | 1999 | 56806.678(55135.256,58394.116) |
|  | 2000 | 56385.634(54689.263,58001.34) |
|  | 2001 | 55895.064(54262.797,57480.45) |
|  | 2002 | 55342.363(53696.497,56910.856) |
|  | 2003 | 54757.162(53154.005,56316.844) |
|  | 2004 | 54174.714(52605.923,55710.18) |
|  | 2005 | 53628.983(52025.093,55176.669) |
|  | 2006 | 53068.221(51540.304,54566.882) |
|  | 2007 | 52455.348(50969.406,53892.989) |
|  | 2008 | 51806.833(50277.276,53237.368) |
|  | 2009 | 51148.882(49622.957,52543.477) |
|  | 2010 | 50538.543(49048.725,51895.517) |
|  | 2011 | 49929.289(48446.588,51258.21) |
|  | 2012 | 49265.74(47888.358,50572.822) |
|  | 2013 | 48592.351(47243.344,49936.106) |
|  | 2014 | 47932.144(46578.309,49299.283) |
|  | 2015 | 47308.635(45983.706,48705.139) |
|  | 2016 | 46645.253(45349.027,48001.153) |
|  | 2017 | 45903.289(44632.898,47252.202) |
|  | 2018 | 45147.334(43858.691,46490.818) |
|  | 2019 | 44434.681(43130.694,45778.152) |
|  | 2020 | 43783.132(42530.911,45120.9) |
|  | 2021 | 43330.832(42150.865,44590.38) |
| Western Pacific | 1990 | 20671.638(20074.281,21298.85) |
|  | 1991 | 20754.463(20146.134,21351.505) |
|  | 1992 | 20802.543(20187.014,21419.533) |
|  | 1993 | 20786.438(20170.529,21437.303) |
|  | 1994 | 20769.267(20134.977,21425.571) |
|  | 1995 | 20790.555(20138.443,21498.667) |
|  | 1996 | 20854.687(20181.008,21558.982) |
|  | 1997 | 20915.305(20234.326,21602.65) |
|  | 1998 | 20930.019(20265.661,21606.946) |
|  | 1999 | 20924.623(20259.722,21610.336) |
|  | 2000 | 20846.781(20157.192,21587.341) |
|  | 2001 | 20721.876(20033.209,21439.554) |
|  | 2002 | 20666.39(19977.857,21344.444) |
|  | 2003 | 20622.859(19896.627,21299.563) |
|  | 2004 | 20510.442(19785.954,21226.951) |
|  | 2005 | 20338.794(19636.66,21078.534) |
|  | 2006 | 20147.632(19485.884,20858.772) |
|  | 2007 | 19940.356(19284.374,20634.169) |
|  | 2008 | 19790.739(19157.789,20482.444) |
|  | 2009 | 19751.778(19119.338,20475.947) |
|  | 2010 | 19938.298(19279.102,20714.433) |
|  | 2011 | 19706(19038.797,20454.392) |
|  | 2012 | 18917.946(18304.382,19584.974) |
|  | 2013 | 18283.271(17702.375,18895.749) |
|  | 2014 | 17678.443(17117.472,18283.785) |
|  | 2015 | 16955.078(16432.788,17533.395) |
|  | 2016 | 16508.585(16035.538,17016.714) |
|  | 2017 | 16489.147(16008.1,17020.109) |
|  | 2018 | 16628.451(16110.564,17205.016) |
|  | 2019 | 16934.854(16360.36,17556.526) |
|  | 2020 | 17342.586(16712.628,18032.027) |
|  | 2021 | 17608.967(16954.317,18344.523) |
| SDI: sociodemographic index; UI: uncertainty interval. | | |

| **Table S2. Trends in the prevalence of neonatal sepsis and other neonatal infections globally and regionally from 1990 to 2021** | | |
| --- | --- | --- |
| Region | Year | Prevalence rate(95%UI) |
| African | 1990 | 902.066(633.61,1285.382) |
|  | 1991 | 901.075(632.59,1281.523) |
|  | 1992 | 900.778(632.373,1279.158) |
|  | 1993 | 900.996(632.637,1278.019) |
|  | 1994 | 901.679(633.235,1277.754) |
|  | 1995 | 903.203(634.39,1278.86) |
|  | 1996 | 906.984(637.384,1282.387) |
|  | 1997 | 913.096(642.148,1289.387) |
|  | 1998 | 919.538(647.103,1298.133) |
|  | 1999 | 924.589(650.931,1304.664) |
|  | 2000 | 926.541(652.213,1306.308) |
|  | 2001 | 923.896(650.481,1301.169) |
|  | 2002 | 917.348(646.003,1290.403) |
|  | 2003 | 908.362(639.34,1275.71) |
|  | 2004 | 898.523(632.088,1260.14) |
|  | 2005 | 889.333(625.202,1246.154) |
|  | 2006 | 880.263(618.917,1232.683) |
|  | 2007 | 870.081(611.997,1216.956) |
|  | 2008 | 859.092(604.674,1200.034) |
|  | 2009 | 847.543(597.545,1182.707) |
|  | 2010 | 835.704(589.514,1165.594) |
|  | 2011 | 821.381(578.493,1145.023) |
|  | 2012 | 803.228(565.042,1119.974) |
|  | 2013 | 782.73(550.024,1091.353) |
|  | 2014 | 761.314(535.473,1059.915) |
|  | 2015 | 740.356(524.656,1026.297) |
|  | 2016 | 715.203(521.609,972.454) |
|  | 2017 | 684.443(502.572,906.778) |
|  | 2018 | 654.536(491.451,866.115) |
|  | 2019 | 631.872(483.093,822.897) |
|  | 2020 | 615.053(475.294,800.401) |
|  | 2021 | 613.181(473.514,797.903) |
| Americas | 1990 | 292.838(212.595,399.027) |
|  | 1991 | 288.849(210.054,393.231) |
|  | 1992 | 285.655(208.079,388.558) |
|  | 1993 | 283.249(206.646,384.975) |
|  | 1994 | 281.539(205.687,382.627) |
|  | 1995 | 280.678(205.224,381.725) |
|  | 1996 | 280.557(205.112,381.938) |
|  | 1997 | 280.872(205.412,382.616) |
|  | 1998 | 281.384(205.895,383.486) |
|  | 1999 | 281.764(206.274,384.172) |
|  | 2000 | 282.139(206.565,384.758) |
|  | 2001 | 282.688(206.987,385.506) |
|  | 2002 | 283.174(207.237,386.215) |
|  | 2003 | 283.555(207.34,386.838) |
|  | 2004 | 283.752(207.312,387.25) |
|  | 2005 | 283.342(206.868,386.883) |
|  | 2006 | 282.947(206.617,386.032) |
|  | 2007 | 283.472(207.031,386.553) |
|  | 2008 | 284.581(207.616,387.975) |
|  | 2009 | 285.557(208.113,389.333) |
|  | 2010 | 285.793(208.101,389.715) |
|  | 2011 | 284.195(206.745,387.803) |
|  | 2012 | 280.814(204.126,383.32) |
|  | 2013 | 276.323(200.836,377.196) |
|  | 2014 | 271.406(197.276,370.14) |
|  | 2015 | 266.825(194.135,363.047) |
|  | 2016 | 260.225(192.989,347.078) |
|  | 2017 | 250.472(189.855,330.181) |
|  | 2018 | 239.934(184.769,314.609) |
|  | 2019 | 231.883(179.987,301.49) |
|  | 2020 | 226.429(178.332,291.373) |
|  | 2021 | 225.168(177.179,289.66) |
| Eastern Mediterranean | 1990 | 616.997(428.923,867.493) |
|  | 1991 | 615.405(428.314,864.832) |
|  | 1992 | 614.216(428.414,862.989) |
|  | 1993 | 613.967(429.245,862.767) |
|  | 1994 | 615.197(431.304,865.101) |
|  | 1995 | 617.046(433.784,868.514) |
|  | 1996 | 621.072(436.932,874.508) |
|  | 1997 | 628.032(442.504,884.736) |
|  | 1998 | 635.756(448.223,896.253) |
|  | 1999 | 642.162(452.951,906.097) |
|  | 2000 | 645.17(455.228,911.151) |
|  | 2001 | 644.186(454.871,908.077) |
|  | 2002 | 640.42(452.327,900.827) |
|  | 2003 | 635.031(448.574,891.652) |
|  | 2004 | 629.745(444.956,883.328) |
|  | 2005 | 625.689(443.125,877.334) |
|  | 2006 | 621.797(440.003,868.667) |
|  | 2007 | 616.796(436.205,858.587) |
|  | 2008 | 611.141(432.498,848.03) |
|  | 2009 | 604.892(428.446,837.579) |
|  | 2010 | 598.069(424.024,826.822) |
|  | 2011 | 588.799(418.105,814.57) |
|  | 2012 | 576.247(409.886,798.129) |
|  | 2013 | 562.048(400.507,779.283) |
|  | 2014 | 547.067(390.911,758.709) |
|  | 2015 | 532.279(380.976,737.268) |
|  | 2016 | 513.633(374.023,688.892) |
|  | 2017 | 489.688(364.28,646.799) |
|  | 2018 | 465.808(354.694,620.62) |
|  | 2019 | 447.516(343.196,588.843) |
|  | 2020 | 434.623(335.79,567.445) |
|  | 2021 | 434.207(335.311,566.551) |
| European | 1990 | 695.1(513.216,938.793) |
|  | 1991 | 685.725(507.175,926.385) |
|  | 1992 | 678.175(502.372,916.291) |
|  | 1993 | 672.893(499.15,909.134) |
|  | 1994 | 668.749(496.632,903.422) |
|  | 1995 | 663.981(493.385,896.735) |
|  | 1996 | 659.203(489.834,890.779) |
|  | 1997 | 656.259(487.701,887.462) |
|  | 1998 | 654.541(486.542,885.912) |
|  | 1999 | 652.956(484.995,884.64) |
|  | 2000 | 652.25(484.139,884.653) |
|  | 2001 | 651.048(483.094,883.142) |
|  | 2002 | 647.666(480.551,878.559) |
|  | 2003 | 641.841(476.191,870.651) |
|  | 2004 | 635.879(471.688,862.655) |
|  | 2005 | 632.639(469.199,858.647) |
|  | 2006 | 631.732(469.201,856.879) |
|  | 2007 | 631.246(469.559,855.707) |
|  | 2008 | 630.743(469.838,854.587) |
|  | 2009 | 628.881(468.981,851.709) |
|  | 2010 | 626.202(467.363,847.888) |
|  | 2011 | 622.243(463.972,841.535) |
|  | 2012 | 614.653(457.727,830.494) |
|  | 2013 | 603.96(449.298,815.344) |
|  | 2014 | 591.918(440.253,798.306) |
|  | 2015 | 579.455(431.468,780.401) |
|  | 2016 | 562.987(426.889,739.869) |
|  | 2017 | 542.839(417.089,706.91) |
|  | 2018 | 524.108(403.877,676.448) |
|  | 2019 | 510.898(399.621,653.554) |
|  | 2020 | 502.356(399.512,640.649) |
|  | 2021 | 499.615(397.931,637.411) |
| Global | 1990 | 598.077(424.852,834.256) |
|  | 1991 | 597.894(425.252,833.685) |
|  | 1992 | 598.393(426.091,834.237) |
|  | 1993 | 599.188(427.09,835.28) |
|  | 1994 | 600.581(428.356,837.153) |
|  | 1995 | 603.013(430.279,840.501) |
|  | 1996 | 606.964(433.048,845.527) |
|  | 1997 | 611.712(436.403,851.522) |
|  | 1998 | 615.865(439.329,856.743) |
|  | 1999 | 619.08(441.557,860.878) |
|  | 2000 | 620.538(442.48,862.816) |
|  | 2001 | 620.072(442.14,861.066) |
|  | 2002 | 618.768(441.276,858.035) |
|  | 2003 | 616.271(439.6,853.512) |
|  | 2004 | 612.445(436.995,847.416) |
|  | 2005 | 608.256(434.109,841.071) |
|  | 2006 | 603.765(431.475,833.515) |
|  | 2007 | 598.687(428.426,824.835) |
|  | 2008 | 594.144(425.719,817.036) |
|  | 2009 | 590.517(423.363,810.928) |
|  | 2010 | 588.764(422.295,807.993) |
|  | 2011 | 582.403(417.809,799.216) |
|  | 2012 | 569.799(408.583,782.313) |
|  | 2013 | 557.4(399.596,765.727) |
|  | 2014 | 544.201(390.599,747.374) |
|  | 2015 | 528.62(380.918,724.485) |
|  | 2016 | 511.494(374.907,689.888) |
|  | 2017 | 494.619(367.992,649.763) |
|  | 2018 | 479.089(362.687,628.515) |
|  | 2019 | 469.307(360.046,609.885) |
|  | 2020 | 463.957(364,600.855) |
|  | 2021 | 465.571(365.167,602.41) |
| High SDI | 1990 | 530.071(385.065,718.079) |
|  | 1991 | 523.784(381.722,709.916) |
|  | 1992 | 517.976(378.704,702.767) |
|  | 1993 | 513.245(376.417,696.92) |
|  | 1994 | 510.041(375.05,693.013) |
|  | 1995 | 507.44(373.428,689.786) |
|  | 1996 | 506.113(372.572,688.812) |
|  | 1997 | 505.522(372.318,688.91) |
|  | 1998 | 504.443(371.71,688.391) |
|  | 1999 | 503.616(371.332,688.293) |
|  | 2000 | 502.335(370.578,687.627) |
|  | 2001 | 500.368(369.117,684.65) |
|  | 2002 | 499.636(368.575,683.288) |
|  | 2003 | 497.864(367.078,680.517) |
|  | 2004 | 493.885(363.901,674.784) |
|  | 2005 | 490.83(361.345,669.856) |
|  | 2006 | 489.981(360.523,668.519) |
|  | 2007 | 489.726(360.238,667.734) |
|  | 2008 | 490.097(360.835,667.808) |
|  | 2009 | 491.014(361.966,668.805) |
|  | 2010 | 495.675(365.789,675.189) |
|  | 2011 | 490.103(361.449,667.601) |
|  | 2012 | 470.58(346.634,641.214) |
|  | 2013 | 452.93(333.297,616.398) |
|  | 2014 | 434.729(319.897,590.795) |
|  | 2015 | 411.645(303.91,558.412) |
|  | 2016 | 391.448(295.461,516.157) |
|  | 2017 | 379.625(291.354,495.889) |
|  | 2018 | 372.011(286.193,481.548) |
|  | 2019 | 371.062(289.114,476.203) |
|  | 2020 | 374.813(296.614,480.101) |
|  | 2021 | 378.611(300.741,484.409) |
| High-middle SDI | 1990 | 289.248(216.309,387.686) |
|  | 1991 | 287.529(215.075,385.354) |
|  | 1992 | 285.777(213.781,383.029) |
|  | 1993 | 283.849(212.329,380.498) |
|  | 1994 | 282.297(211.147,378.493) |
|  | 1995 | 281.369(210.65,377.361) |
|  | 1996 | 280.559(210.098,376.102) |
|  | 1997 | 279.793(209.595,374.906) |
|  | 1998 | 279.058(209.044,373.711) |
|  | 1999 | 278.172(208.361,372.264) |
|  | 2000 | 277.133(207.556,370.63) |
|  | 2001 | 275.705(206.454,368.496) |
|  | 2002 | 274.202(205.362,366.197) |
|  | 2003 | 272.672(204.294,363.825) |
|  | 2004 | 271.506(203.425,361.95) |
|  | 2005 | 270.316(202.444,360.217) |
|  | 2006 | 269.5(201.954,358.98) |
|  | 2007 | 269.808(202.306,359.241) |
|  | 2008 | 270.774(203.138,360.509) |
|  | 2009 | 271.792(203.995,361.902) |
|  | 2010 | 272.527(204.607,362.973) |
|  | 2011 | 272.552(204.754,363.05) |
|  | 2012 | 271.666(204.194,361.906) |
|  | 2013 | 270.261(203.283,360.083) |
|  | 2014 | 268.741(202.408,358.074) |
|  | 2015 | 266.609(201.27,354.917) |
|  | 2016 | 261.957(199.294,342.471) |
|  | 2017 | 254.479(197.478,330.94) |
|  | 2018 | 246.735(193.413,317.977) |
|  | 2019 | 241.095(190.347,307.502) |
|  | 2020 | 237.256(189.822,301.919) |
|  | 2021 | 236.442(189.425,300.999) |
| Low SDI | 1990 | 750.392(523.876,1063.78) |
|  | 1991 | 744.5(521.152,1055.131) |
|  | 1992 | 739.453(518.889,1047.757) |
|  | 1993 | 735.28(517.088,1041.655) |
|  | 1994 | 732.001(515.778,1036.741) |
|  | 1995 | 729.759(514.363,1033.162) |
|  | 1996 | 728.899(514.615,1030.64) |
|  | 1997 | 729.377(515.849,1029.492) |
|  | 1998 | 730.172(517.348,1028.82) |
|  | 1999 | 730.267(518.27,1027.561) |
|  | 2000 | 728.801(517.752,1024.734) |
|  | 2001 | 725.099(515.732,1017.292) |
|  | 2002 | 719.67(512.631,1007.554) |
|  | 2003 | 713.128(508.765,996.585) |
|  | 2004 | 706.504(504.451,985.875) |
|  | 2005 | 700.849(500.743,976.869) |
|  | 2006 | 695.265(497.427,967.248) |
|  | 2007 | 688.686(492.826,955.863) |
|  | 2008 | 681.473(487.426,943.79) |
|  | 2009 | 673.957(481.86,932.191) |
|  | 2010 | 666.548(476.403,921.121) |
|  | 2011 | 658.089(470.797,909.236) |
|  | 2012 | 647.688(463.498,895.463) |
|  | 2013 | 636.153(455.354,879.973) |
|  | 2014 | 624.028(447.062,862.944) |
|  | 2015 | 611.904(439.151,844.594) |
|  | 2016 | 595.123(435.453,804.736) |
|  | 2017 | 572.377(424.69,750.863) |
|  | 2018 | 549.619(413.932,726.056) |
|  | 2019 | 532.683(406.876,693.433) |
|  | 2020 | 521.337(405.186,671.526) |
|  | 2021 | 519.798(405.53,667.694) |
| Low-middle SDI | 1990 | 850.097(593.411,1216.113) |
|  | 1991 | 848.494(592.675,1210.739) |
|  | 1992 | 846.892(592.16,1206.016) |
|  | 1993 | 845.045(591.435,1201.597) |
|  | 1994 | 843.775(591.091,1198.507) |
|  | 1995 | 844.009(591.769,1199.206) |
|  | 1996 | 846.758(593.785,1201.789) |
|  | 1997 | 851.563(597.285,1206.669) |
|  | 1998 | 856.59(601.053,1211.33) |
|  | 1999 | 860.525(604.065,1214.657) |
|  | 2000 | 862.22(605.369,1215.348) |
|  | 2001 | 859.839(603.165,1210.874) |
|  | 2002 | 852.979(597.774,1199.917) |
|  | 2003 | 843.411(590.333,1185.286) |
|  | 2004 | 833.666(582.868,1170.699) |
|  | 2005 | 825.247(576.647,1158.308) |
|  | 2006 | 817.177(572.432,1147.211) |
|  | 2007 | 808.306(567.644,1133.661) |
|  | 2008 | 798.878(562.438,1118.823) |
|  | 2009 | 788.847(556.747,1103.564) |
|  | 2010 | 778.743(550.805,1088.888) |
|  | 2011 | 766.689(541.314,1072.046) |
|  | 2012 | 751.299(529.249,1051.195) |
|  | 2013 | 733.92(515.877,1027.24) |
|  | 2014 | 715.483(502.18,1000.39) |
|  | 2015 | 696.962(489.985,970.853) |
|  | 2016 | 673.559(489.175,920.459) |
|  | 2017 | 643.957(471.108,851.794) |
|  | 2018 | 614.888(460.759,811.889) |
|  | 2019 | 593.031(452.438,774.511) |
|  | 2020 | 577.543(446.135,754.034) |
|  | 2021 | 576.142(444.772,749.604) |
| Middle SDI | 1990 | 448.917(322.548,619.794) |
|  | 1991 | 450.327(323.74,621.081) |
|  | 1992 | 451.913(324.977,622.633) |
|  | 1993 | 453.241(325.945,623.884) |
|  | 1994 | 454.976(327.109,625.807) |
|  | 1995 | 457.749(328.941,629.355) |
|  | 1996 | 461.936(332.172,634.866) |
|  | 1997 | 466.205(335.443,640.45) |
|  | 1998 | 469.501(337.473,644.732) |
|  | 1999 | 472.152(339.013,648.215) |
|  | 2000 | 473.303(339.485,649.706) |
|  | 2001 | 473.73(339.804,649.867) |
|  | 2002 | 475.371(341.032,651.935) |
|  | 2003 | 477.046(342.291,654.055) |
|  | 2004 | 477.007(342.303,653.865) |
|  | 2005 | 475.533(341.171,651.769) |
|  | 2006 | 473.03(339.453,647.982) |
|  | 2007 | 469.423(336.963,642.573) |
|  | 2008 | 466.609(335.062,638.337) |
|  | 2009 | 465.665(334.512,636.82) |
|  | 2010 | 468.08(336.346,639.992) |
|  | 2011 | 464.236(333.672,634.168) |
|  | 2012 | 452.354(325.127,617.576) |
|  | 2013 | 442.248(318.004,603.451) |
|  | 2014 | 431.42(310.814,588.143) |
|  | 2015 | 416.612(301.528,567.275) |
|  | 2016 | 402.158(296.826,536.868) |
|  | 2017 | 391.804(295.613,514.473) |
|  | 2018 | 383.381(294.032,498.846) |
|  | 2019 | 379.684(295.816,489.752) |
|  | 2020 | 379.586(296.969,489.186) |
|  | 2021 | 382.344(299.203,491.947) |
| South-East Asia | 1990 | 779.424(543.463,1101.689) |
|  | 1991 | 771.881(539.601,1090.663) |
|  | 1992 | 764.886(535.945,1080.579) |
|  | 1993 | 758.549(532.58,1071.592) |
|  | 1994 | 753.09(529.683,1063.953) |
|  | 1995 | 748.787(527.471,1058.101) |
|  | 1996 | 745.083(525.602,1051.592) |
|  | 1997 | 741.418(523.756,1044.638) |
|  | 1998 | 737.539(521.647,1037.253) |
|  | 1999 | 733.413(518.819,1029.699) |
|  | 2000 | 729.082(515.769,1022.27) |
|  | 2001 | 723.663(512.383,1012.089) |
|  | 2002 | 716.994(508.112,1000.182) |
|  | 2003 | 709.701(503.397,987.489) |
|  | 2004 | 702.524(498.768,975.117) |
|  | 2005 | 696.229(494.653,965.094) |
|  | 2006 | 689.794(491.22,955.328) |
|  | 2007 | 682.4(487.011,944.124) |
|  | 2008 | 674.535(482.403,932.367) |
|  | 2009 | 666.801(477.752,920.991) |
|  | 2010 | 660.126(473.556,911.221) |
|  | 2011 | 653.782(469.058,900.129) |
|  | 2012 | 646.642(463.675,890.325) |
|  | 2013 | 639.068(457.94,880.095) |
|  | 2014 | 631.249(452.25,868.925) |
|  | 2015 | 623.226(446.978,855.537) |
|  | 2016 | 609.9(444.905,818.265) |
|  | 2017 | 590.016(439.615,770.662) |
|  | 2018 | 569.514(430.709,743.901) |
|  | 2019 | 554.243(427.51,717.45) |
|  | 2020 | 545.126(424.313,701.226) |
|  | 2021 | 543.094(423.93,694.761) |
| Western Pacific | 1990 | 269.425(194.592,370.26) |
|  | 1991 | 270.894(196.156,371.965) |
|  | 1992 | 271.87(197.012,373.099) |
|  | 1993 | 272.004(197.149,373.157) |
|  | 1994 | 272.145(197.558,373.273) |
|  | 1995 | 272.793(198.316,374.138) |
|  | 1996 | 273.928(198.779,375.869) |
|  | 1997 | 274.876(199.058,377.9) |
|  | 1998 | 275.159(198.817,379.289) |
|  | 1999 | 275.215(198.727,380.369) |
|  | 2000 | 274.428(198.321,380.235) |
|  | 2001 | 273.143(197.342,378.398) |
|  | 2002 | 272.845(197.063,377.919) |
|  | 2003 | 272.75(196.905,377.737) |
|  | 2004 | 271.801(196.131,375.847) |
|  | 2005 | 270.15(194.856,373.071) |
|  | 2006 | 268.213(193.423,370.385) |
|  | 2007 | 265.925(191.802,367.119) |
|  | 2008 | 264.275(190.68,364.874) |
|  | 2009 | 263.993(190.784,364.772) |
|  | 2010 | 266.597(192.855,368.757) |
|  | 2011 | 263.804(190.971,364.76) |
|  | 2012 | 253.892(183.891,350.609) |
|  | 2013 | 245.839(178.064,339.122) |
|  | 2014 | 237.797(172.394,327.619) |
|  | 2015 | 227.756(165.753,312.536) |
|  | 2016 | 220.039(162.832,294.508) |
|  | 2017 | 216.548(163.3,286.848) |
|  | 2018 | 214.955(166.624,282.064) |
|  | 2019 | 216.718(168.999,280.888) |
|  | 2020 | 221.086(172.658,284.362) |
|  | 2021 | 225.27(175.672,290.252) |
| SDI: sociodemographic index; UI: uncertainty interval. | | |

| **Table S3. Trends in the DALYs of neonatal sepsis and other neonatal infections globally and regionally from 1990 to 2021** | | |
| --- | --- | --- |
| Region | Year | DALYs rate(95%UI) |
| African | 1990 | 520318.567(446182.668,587634.89) |
|  | 1991 | 515395.649(444332.653,581494.004) |
|  | 1992 | 509848.695(437076.643,575558.64) |
|  | 1993 | 505874.619(432132.144,575211.894) |
|  | 1994 | 503603.756(429140.284,573794.957) |
|  | 1995 | 500886.186(426686.438,574126.745) |
|  | 1996 | 496687.085(421652.772,572069.771) |
|  | 1997 | 493094.396(416578.548,569913.32) |
|  | 1998 | 490576.533(414089.599,568652.982) |
|  | 1999 | 486346.248(411069.094,564279.63) |
|  | 2000 | 481598.856(408620.014,559089.144) |
|  | 2001 | 476713.921(406541.208,554852.846) |
|  | 2002 | 473852.447(403157.05,553232.326) |
|  | 2003 | 470841.558(400294.802,550032.265) |
|  | 2004 | 467760.554(398571.276,545385.818) |
|  | 2005 | 465338.512(399220.451,542150.159) |
|  | 2006 | 461424.994(396572.174,540230.126) |
|  | 2007 | 457110.341(391855.805,533199.355) |
|  | 2008 | 451256.379(388076.53,525314.615) |
|  | 2009 | 444992.738(383145.805,519324.658) |
|  | 2010 | 438962.283(378890.801,510982) |
|  | 2011 | 435054.961(374514.191,510003.921) |
|  | 2012 | 432552.384(371397.797,506388.357) |
|  | 2013 | 429558.39(367562.773,498457.465) |
|  | 2014 | 425731.428(362527.849,491774.624) |
|  | 2015 | 419422.27(356164.484,483979.223) |
|  | 2016 | 412619.531(348471.896,478412.221) |
|  | 2017 | 402493.846(334339.88,474340.078) |
|  | 2018 | 393932.386(325263.403,470203.311) |
|  | 2019 | 388647.97(321454.451,467022.353) |
|  | 2020 | 376059.945(310810.291,452917.141) |
|  | 2021 | 367540.097(302629.912,446330.433) |
| Americas | 1990 | 171864.702(159718.84,185504.369) |
|  | 1991 | 171231.818(158976.215,185046.113) |
|  | 1992 | 169418.086(156556.062,183843.296) |
|  | 1993 | 169147.173(157002.606,182538.035) |
|  | 1994 | 169308.352(158602.348,182055.279) |
|  | 1995 | 170931.472(159107.157,183607.131) |
|  | 1996 | 173736.237(162065.134,186285.787) |
|  | 1997 | 174214.008(162348.29,186960.55) |
|  | 1998 | 171686.29(160140.878,184619.004) |
|  | 1999 | 169046.024(156559.083,181919.851) |
|  | 2000 | 168096.729(156941.402,180891.523) |
|  | 2001 | 167247.963(155574.387,181184.257) |
|  | 2002 | 163584.043(151837.182,177417.544) |
|  | 2003 | 161843.636(150395.468,175179.574) |
|  | 2004 | 160962.128(149181.266,173918.439) |
|  | 2005 | 157707.866(145946.683,171530.587) |
|  | 2006 | 154183.53(142016.495,167712.304) |
|  | 2007 | 152677.281(139418.694,167400.871) |
|  | 2008 | 149863.043(135553.048,164513.289) |
|  | 2009 | 147263.108(132708.942,162198.718) |
|  | 2010 | 142095.139(128119.539,157053.989) |
|  | 2011 | 140117.337(125201.164,155005.89) |
|  | 2012 | 136314.327(122288.74,151663.369) |
|  | 2013 | 132008.605(117977.542,147563.292) |
|  | 2014 | 128403.674(113710.975,144456.95) |
|  | 2015 | 125205.271(110152.809,141637.912) |
|  | 2016 | 122333.163(107356.002,140002.6) |
|  | 2017 | 119753.319(103908.38,138833.734) |
|  | 2018 | 116795.404(99911.94,136997.878) |
|  | 2019 | 113993.243(96198.776,135083.808) |
|  | 2020 | 106964.375(88457.768,127299.844) |
|  | 2021 | 103016.42(82065.742,125631.957) |
| Eastern Mediterranean | 1990 | 151092.702(121449.921,189657.509) |
|  | 1991 | 152342.094(122902.508,188375.751) |
|  | 1992 | 152969.204(123808.981,189516.22) |
|  | 1993 | 153355.03(123084.524,191348.772) |
|  | 1994 | 154738.52(124959.56,193796.957) |
|  | 1995 | 156925.511(126695.633,196129.712) |
|  | 1996 | 157642.66(129357.211,196353.214) |
|  | 1997 | 157764.701(128496.16,199485.265) |
|  | 1998 | 156566.189(127940.259,200117.512) |
|  | 1999 | 155828.803(127481.198,197593.882) |
|  | 2000 | 154472.571(125072.003,191921.984) |
|  | 2001 | 153160.531(123996.382,190317.443) |
|  | 2002 | 152350.742(124665.721,186698.24) |
|  | 2003 | 151479.08(124193.969,184016.809) |
|  | 2004 | 150351.936(124157.456,182950.302) |
|  | 2005 | 148041.739(122570.423,179454.89) |
|  | 2006 | 145843.427(121663.501,175323.073) |
|  | 2007 | 142470.268(118400.796,173717.844) |
|  | 2008 | 139642.806(116084.673,167668.748) |
|  | 2009 | 136688.775(113586.764,161540.695) |
|  | 2010 | 134330.582(111328.474,162917.511) |
|  | 2011 | 132119.622(108713.014,159550.64) |
|  | 2012 | 130487.936(105740.874,159427.61) |
|  | 2013 | 130028.13(104623.2,161293.399) |
|  | 2014 | 129755.526(102514.05,160648.107) |
|  | 2015 | 128749.986(100974.47,159807.804) |
|  | 2016 | 127837.984(99640.201,162255.904) |
|  | 2017 | 127251.448(98498.023,161436.05) |
|  | 2018 | 125896.438(96967.283,159974.28) |
|  | 2019 | 126937.01(98142.36,159805.191) |
|  | 2020 | 133062.374(103684.889,167875.53) |
|  | 2021 | 131698.767(101946.841,164956.965) |
| European | 1990 | 55244.457(48363.584,63440.904) |
|  | 1991 | 55200.431(48199.006,63845.108) |
|  | 1992 | 53877.981(46647.913,62829.441) |
|  | 1993 | 53452.709(46467.651,62442.761) |
|  | 1994 | 53100.795(46016.118,61493.931) |
|  | 1995 | 52905.774(46253.484,61129.632) |
|  | 1996 | 52189.964(45831.682,60033.167) |
|  | 1997 | 51987.344(45785.206,59452.857) |
|  | 1998 | 51807.364(45899.787,59170.334) |
|  | 1999 | 52339.437(46654.215,59979.16) |
|  | 2000 | 51619.213(46120.241,59353.327) |
|  | 2001 | 50254.848(44882.302,57667.034) |
|  | 2002 | 48325.327(43266.208,55147.646) |
|  | 2003 | 47043.86(41891.339,53330.263) |
|  | 2004 | 45199.602(40511.815,50717.337) |
|  | 2005 | 43622.475(39191.48,48807.454) |
|  | 2006 | 42528.89(38161.757,47384.02) |
|  | 2007 | 41399.394(37380.336,46053.324) |
|  | 2008 | 40573.237(36819.164,44696.218) |
|  | 2009 | 39705.925(36098.799,43506.796) |
|  | 2010 | 38322.774(34966.696,41999.261) |
|  | 2011 | 38669.659(35470.404,41764.883) |
|  | 2012 | 39843.373(36745.227,43020.991) |
|  | 2013 | 39515.145(36515.143,42575.248) |
|  | 2014 | 39459.888(36352.59,42545.65) |
|  | 2015 | 37184.203(34271.364,40261.909) |
|  | 2016 | 35996.122(33036.757,39248.399) |
|  | 2017 | 36128.707(33029.861,39779.302) |
|  | 2018 | 35625.161(32264.215,39515.299) |
|  | 2019 | 35443.057(31681.943,39711.075) |
|  | 2020 | 33221.064(29105.058,37801.455) |
|  | 2021 | 32898.373(28407.586,37991.795) |
| Global | 1990 | 238416.27(210446.838,265861.961) |
|  | 1991 | 240004.749(213383.206,268199.989) |
|  | 1992 | 240967.811(215053.508,268985.3) |
|  | 1993 | 242056.904(217498.974,269255.99) |
|  | 1994 | 243060.265(217209.428,271501.325) |
|  | 1995 | 244136.234(217363.555,273272.921) |
|  | 1996 | 244950.813(219441.202,273781.882) |
|  | 1997 | 244929.505(218918.367,272598.394) |
|  | 1998 | 244916.618(219062.906,272077.811) |
|  | 1999 | 244464.986(218873.565,272659.452) |
|  | 2000 | 242680.845(217517.924,271411.015) |
|  | 2001 | 240590.62(215324.191,269639.837) |
|  | 2002 | 238473.033(213528.6,267594.514) |
|  | 2003 | 237143.1(210923.416,267379.838) |
|  | 2004 | 235759.197(209123.076,267058.275) |
|  | 2005 | 233256.702(207402.896,263270.135) |
|  | 2006 | 228517.814(204220.373,258873.076) |
|  | 2007 | 224087.336(199608.236,253582.512) |
|  | 2008 | 219799.745(196052.905,247491.715) |
|  | 2009 | 217318.796(194104.488,242181.676) |
|  | 2010 | 215396.518(192171.328,241812.115) |
|  | 2011 | 213669.308(190708.54,239785.153) |
|  | 2012 | 210338.229(187176.747,236903.817) |
|  | 2013 | 208324.213(183765.223,234528.511) |
|  | 2014 | 205781.312(179910.116,232622.305) |
|  | 2015 | 201213.651(174968.755,229224.483) |
|  | 2016 | 196734.557(169196.472,226216.706) |
|  | 2017 | 194500.822(165833.292,224804.234) |
|  | 2018 | 193306.215(165108.958,223062.576) |
|  | 2019 | 194757.634(166628.478,226931.722) |
|  | 2020 | 192856.441(164382.928,225772.001) |
|  | 2021 | 190657.6(161129.49,224146.627) |
| High SDI | 1990 | 65420.705(57992.65,73322.98) |
|  | 1991 | 66015.103(58502.254,73834.488) |
|  | 1992 | 65735.389(58191.587,73995.815) |
|  | 1993 | 65570.362(57937.922,73763.264) |
|  | 1994 | 65172.528(57366.664,73340.967) |
|  | 1995 | 65321.818(57489.211,73157.766) |
|  | 1996 | 65065.398(57842.406,72849.749) |
|  | 1997 | 64731.032(57693.622,72169.193) |
|  | 1998 | 64259.987(57871.558,71492.197) |
|  | 1999 | 63748.806(57467.633,70915.968) |
|  | 2000 | 61791.888(55999.879,69048.553) |
|  | 2001 | 59931.819(54377.985,66585.494) |
|  | 2002 | 58818.746(53243.008,65229.898) |
|  | 2003 | 57549.094(52195.416,63708.711) |
|  | 2004 | 54828.069(49617.854,60972.719) |
|  | 2005 | 51862.517(46984.059,57728.299) |
|  | 2006 | 49065.908(44484.687,54009.998) |
|  | 2007 | 47305.628(42996.12,52489.726) |
|  | 2008 | 46343.779(42167.011,51242.231) |
|  | 2009 | 45759.535(41655.77,50874.828) |
|  | 2010 | 45051.135(41153.801,50166.948) |
|  | 2011 | 44778.115(41120.662,49479.412) |
|  | 2012 | 43975.172(40213.993,48688.852) |
|  | 2013 | 42318.147(38776.176,46950.528) |
|  | 2014 | 41693.062(38233.211,45635.49) |
|  | 2015 | 39179.651(35805.535,43187.505) |
|  | 2016 | 37371.987(34005.281,41146.655) |
|  | 2017 | 36719.484(33284.641,40811.021) |
|  | 2018 | 35940.457(32380.357,40103.225) |
|  | 2019 | 36039.358(32141.296,40882.492) |
|  | 2020 | 34445.361(29904.372,39569.54) |
|  | 2021 | 34296.026(29133.468,39971.304) |
| High-middle SDI | 1990 | 30810.054(27660.359,34528.896) |
|  | 1991 | 30360.438(27244.204,34075.813) |
|  | 1992 | 29691.514(26638.437,33232.528) |
|  | 1993 | 28824.072(25747.905,32280.853) |
|  | 1994 | 28792.086(25786.314,32046.99) |
|  | 1995 | 28094.236(25231.765,31166.466) |
|  | 1996 | 27293.27(24538.76,30200.211) |
|  | 1997 | 26836.909(24044.848,29828.61) |
|  | 1998 | 26370.508(23556.493,29277.667) |
|  | 1999 | 26286.733(23520.588,29040.725) |
|  | 2000 | 26024.377(23382.448,28766.481) |
|  | 2001 | 25239.902(22815.8,27765.559) |
|  | 2002 | 24956.492(22581.104,27312.647) |
|  | 2003 | 24646.884(22610.797,26676.911) |
|  | 2004 | 24131.243(22324.603,25906.02) |
|  | 2005 | 23303.559(21607.139,24979.47) |
|  | 2006 | 21996.902(20529.679,23481.802) |
|  | 2007 | 21129.624(19781.425,22555.867) |
|  | 2008 | 19865.894(18706.167,21035.651) |
|  | 2009 | 18359.597(17305.725,19450.838) |
|  | 2010 | 17082.076(16030.951,18014.715) |
|  | 2011 | 16145.797(15230.89,16934.926) |
|  | 2012 | 15817.417(14954.391,16658.54) |
|  | 2013 | 15416.11(14523.606,16393.17) |
|  | 2014 | 15337.102(14500.913,16319.71) |
|  | 2015 | 15408.01(14545.624,16363.316) |
|  | 2016 | 15338.692(14414.646,16331.786) |
|  | 2017 | 15430.763(14458.875,16482.813) |
|  | 2018 | 15239.55(14218.004,16312.31) |
|  | 2019 | 15353.541(14244.63,16488.587) |
|  | 2020 | 14576.347(13312.442,15817.316) |
|  | 2021 | 14038.304(12386.934,15556.5) |
| Low SDI | 1990 | 348555.327(300930.352,400719.229) |
|  | 1991 | 346133.168(299393.169,397154.294) |
|  | 1992 | 343201.965(299521.953,395194.3) |
|  | 1993 | 340568.942(296012.281,389629.04) |
|  | 1994 | 337631.28(289744.759,385543.18) |
|  | 1995 | 333984.103(289316.622,383539.855) |
|  | 1996 | 330382.572(286700.37,378251.448) |
|  | 1997 | 325545.993(283992.739,369138.452) |
|  | 1998 | 322287.5(284709.11,363613.481) |
|  | 1999 | 319263.204(280450.101,357234.678) |
|  | 2000 | 313999.836(277758.39,352725.277) |
|  | 2001 | 308298.36(274205.378,344680.056) |
|  | 2002 | 301760.965(268377.301,338893.367) |
|  | 2003 | 297273.666(262647.099,334780.865) |
|  | 2004 | 293753.671(257901.956,331568.794) |
|  | 2005 | 288648.301(253834.431,328811.515) |
|  | 2006 | 280826.71(248643.324,319418.328) |
|  | 2007 | 273236.711(241042.81,310182.006) |
|  | 2008 | 265786.532(233497.673,304252.202) |
|  | 2009 | 259930.25(226263.036,296136.774) |
|  | 2010 | 253490.623(221674.762,292181.423) |
|  | 2011 | 248983.16(215945.506,289906.696) |
|  | 2012 | 244947.693(210644.17,286226.843) |
|  | 2013 | 243697.758(210025.106,286846.993) |
|  | 2014 | 241357.192(207207.515,283777.709) |
|  | 2015 | 238477.52(203205.459,282887.307) |
|  | 2016 | 233489.928(197202.545,279378.261) |
|  | 2017 | 228021.88(190380.839,275488.353) |
|  | 2018 | 222702.704(183796.591,266926.081) |
|  | 2019 | 220735.477(181291.308,264626.844) |
|  | 2020 | 216729.917(177304.919,259538.897) |
|  | 2021 | 210808.301(170917.501,259712.9) |
| Low-middle SDI | 1990 | 482534.033(421173.967,551486.224) |
|  | 1991 | 479160.606(419352.458,550274.934) |
|  | 1992 | 473880.252(413028.205,542980.728) |
|  | 1993 | 468945.421(407790.217,541073.873) |
|  | 1994 | 465136.719(402060.504,540473.041) |
|  | 1995 | 460905.407(394122.146,541213.829) |
|  | 1996 | 455296.461(385717.338,538751.243) |
|  | 1997 | 450349.323(378507.648,534859.253) |
|  | 1998 | 447340.388(378069.67,529921.836) |
|  | 1999 | 443198.612(374423.073,524743.331) |
|  | 2000 | 438593.462(370604.347,521011.806) |
|  | 2001 | 434524.897(367985.649,519111.103) |
|  | 2002 | 431667.771(362691.78,520051.216) |
|  | 2003 | 428887.576(362999.037,521465.982) |
|  | 2004 | 426767.87(361134.052,518666.7) |
|  | 2005 | 425377.742(361725.939,516060.793) |
|  | 2006 | 420661.753(358289.636,512270.646) |
|  | 2007 | 416433.85(356103.145,506191.859) |
|  | 2008 | 410922.291(351368.86,497709.938) |
|  | 2009 | 406405.039(349549.328,490376.166) |
|  | 2010 | 402039.643(345567.867,485529.187) |
|  | 2011 | 399929.527(343034.486,484415.349) |
|  | 2012 | 398654.732(338315.481,483486.758) |
|  | 2013 | 396945.625(336937.26,475390.013) |
|  | 2014 | 394117.616(336249.867,470893.617) |
|  | 2015 | 389117.409(331487.836,460739.091) |
|  | 2016 | 382982.735(322383.095,452929.27) |
|  | 2017 | 374365.746(312221.634,445770.297) |
|  | 2018 | 366728.415(301999.561,442951.573) |
|  | 2019 | 361921.452(294122.508,442699.712) |
|  | 2020 | 351954.93(285906.491,430655.947) |
|  | 2021 | 343708.076(277975.551,423698.816) |
| Middle SDI | 1990 | 147977.628(127616.987,172488.862) |
|  | 1991 | 149075.772(129158.004,174759.025) |
|  | 1992 | 149546.485(130388.51,174574.703) |
|  | 1993 | 150343.088(130734.905,175491.551) |
|  | 1994 | 150996.424(132377.426,174797.081) |
|  | 1995 | 153064.16(134298.122,176316.54) |
|  | 1996 | 155411.125(136985.97,178997.315) |
|  | 1997 | 156422.129(139570.549,178477.442) |
|  | 1998 | 156017.05(139543.224,178227.551) |
|  | 1999 | 155716.363(139129.463,177562.598) |
|  | 2000 | 155422.991(139218.862,177415.959) |
|  | 2001 | 154617.657(138334.544,177885.844) |
|  | 2002 | 152537.252(136592.879,176867.671) |
|  | 2003 | 151207.166(134789.682,175263.411) |
|  | 2004 | 150254.597(132630.03,174460.856) |
|  | 2005 | 148417.684(131233.578,172218.522) |
|  | 2006 | 145416.282(129359.272,169195.212) |
|  | 2007 | 142442.843(126297.724,165481.116) |
|  | 2008 | 139139.7(122977.436,162540.118) |
|  | 2009 | 137638.809(120458.125,161644.705) |
|  | 2010 | 136321.886(119264.068,161215.739) |
|  | 2011 | 134783.878(117772.405,159544.134) |
|  | 2012 | 130128.23(112730.307,154269.086) |
|  | 2013 | 126330.419(107397.988,152049.175) |
|  | 2014 | 122527.9(104201.044,147928.204) |
|  | 2015 | 116991.598(99201.294,143229.067) |
|  | 2016 | 111842.567(94504.505,136799.22) |
|  | 2017 | 110753.492(92999.852,135271.313) |
|  | 2018 | 110750.132(91610.861,135474.989) |
|  | 2019 | 112350.528(92017.175,137651.37) |
|  | 2020 | 110190.294(91964.458,133859.97) |
|  | 2021 | 109752.871(90593.928,133588.595) |
| South-East Asia | 1990 | 324915.372(279485.185,378239.612) |
|  | 1991 | 322920.35(279514.437,375318.548) |
|  | 1992 | 319963.941(275188.276,371548.592) |
|  | 1993 | 316907.514(271483.1,366701.071) |
|  | 1994 | 313085.037(267824.96,361110.464) |
|  | 1995 | 308052.537(263065.495,358009.138) |
|  | 1996 | 302613.041(258761.451,349929.188) |
|  | 1997 | 296020.737(255261.077,339846.482) |
|  | 1998 | 292573.431(255170.12,333645.383) |
|  | 1999 | 289207.795(253128.826,329747.959) |
|  | 2000 | 283105.911(247532.995,324279.126) |
|  | 2001 | 277041.602(244075.961,318665.331) |
|  | 2002 | 269840.036(237331.261,307916.955) |
|  | 2003 | 264676.122(231457.625,305296.023) |
|  | 2004 | 261260.389(226617.891,303359.615) |
|  | 2005 | 256574.788(220724.516,301323.502) |
|  | 2006 | 246428.019(212566.681,291443.74) |
|  | 2007 | 237686.956(204348.274,277802.326) |
|  | 2008 | 228785.129(195341.457,266669.867) |
|  | 2009 | 223983.924(191089.837,262199.58) |
|  | 2010 | 218546.552(185268.884,255048.764) |
|  | 2011 | 215051.336(182211.136,251821.243) |
|  | 2012 | 211435.366(178285.051,251496.408) |
|  | 2013 | 210399(177748.118,251081.991) |
|  | 2014 | 207723.705(173644.987,246553.68) |
|  | 2015 | 205667.509(173371.621,249666.213) |
|  | 2016 | 199953.431(167027.964,240603.598) |
|  | 2017 | 195576.608(161130.823,235856.372) |
|  | 2018 | 191737.803(157736.75,234346.76) |
|  | 2019 | 190378.832(155260.466,230491.564) |
|  | 2020 | 186081.992(149507.832,225627.15) |
|  | 2021 | 180599.793(144573.244,220468.12) |
| Western Pacific | 1990 | 79573.43(67402.795,92759.458) |
|  | 1991 | 80187.909(68829.653,93631.448) |
|  | 1992 | 80942.315(69780.457,94117.831) |
|  | 1993 | 81344.156(70184.399,94787.882) |
|  | 1994 | 81190.016(70734.097,93913.939) |
|  | 1995 | 81700.801(70611.047,94624.806) |
|  | 1996 | 82416.574(71842.296,95450.301) |
|  | 1997 | 82544.735(72024.694,94657.123) |
|  | 1998 | 81787.173(71279.846,92798.721) |
|  | 1999 | 81763.591(71565.266,92999.509) |
|  | 2000 | 81549.789(70965.47,92197.802) |
|  | 2001 | 80561.129(69284.607,92383.506) |
|  | 2002 | 79560.004(69093.033,91413.693) |
|  | 2003 | 78771.24(68971.055,89404.654) |
|  | 2004 | 77096.363(67583.636,87739.99) |
|  | 2005 | 75103.166(65330.205,85654.807) |
|  | 2006 | 73066.86(63750.65,83653.151) |
|  | 2007 | 71182.872(61959.115,81261.261) |
|  | 2008 | 70536.953(61631.723,80348.166) |
|  | 2009 | 70980.161(62209.437,80787.345) |
|  | 2010 | 72396.902(63116.817,82620.083) |
|  | 2011 | 71518.298(62964.638,82482.108) |
|  | 2012 | 66954.875(58567.934,77321.414) |
|  | 2013 | 63921.228(55299.057,73276.1) |
|  | 2014 | 61360.911(53246.419,69863.811) |
|  | 2015 | 56682.367(48820.446,64763.976) |
|  | 2016 | 53165.449(45562.547,61094.474) |
|  | 2017 | 52833.042(44725.479,61626.724) |
|  | 2018 | 53200.966(44746.915,61973.204) |
|  | 2019 | 55861.029(46860.944,65980.182) |
|  | 2020 | 55809.6(47274.388,66129.657) |
|  | 2021 | 57179.586(48050.587,67524.519) |
| DALY: disability adjust life year; SDI: sociodemographic index; UI: uncertainty interval. | | |

| **Table S4. Trends in the death of neonatal sepsis and other neonatal infections globally and regionally from 1990 to 2021** | | |
| --- | --- | --- |
| Region | Year | death rate(95%UI) |
| African | 1990 | 5781.723(4957.8,6530.044) |
|  | 1991 | 5727.014(4936.466,6461.676) |
|  | 1992 | 5665.367(4855.982,6395.771) |
|  | 1993 | 5621.201(4801.419,6391.78) |
|  | 1994 | 5595.964(4768.09,6376.477) |
|  | 1995 | 5565.76(4740.999,6379.725) |
|  | 1996 | 5519.088(4685.126,6357.102) |
|  | 1997 | 5479.155(4629.089,6332.871) |
|  | 1998 | 5451.165(4600.918,6318.687) |
|  | 1999 | 5404.146(4567.684,6270.071) |
|  | 2000 | 5351.382(4540.521,6212.911) |
|  | 2001 | 5297.094(4516.933,6165.394) |
|  | 2002 | 5265.3(4479.506,6147.382) |
|  | 2003 | 5231.85(4447.541,6112.099) |
|  | 2004 | 5197.621(4428.351,6059.892) |
|  | 2005 | 5170.714(4436.038,6024.341) |
|  | 2006 | 5127.234(4406.383,6003.086) |
|  | 2007 | 5079.296(4354.268,5925.009) |
|  | 2008 | 5014.25(4312.062,5836.65) |
|  | 2009 | 4944.654(4257.069,5770.115) |
|  | 2010 | 4877.648(4210.046,5677.835) |
|  | 2011 | 4834.24(4161.318,5667.199) |
|  | 2012 | 4806.449(4127.049,5627.165) |
|  | 2013 | 4773.199(4084.2,5539.027) |
|  | 2014 | 4730.694(4028.382,5464.592) |
|  | 2015 | 4660.604(3957.382,5378.29) |
|  | 2016 | 4585.045(3871.921,5316.307) |
|  | 2017 | 4472.571(3715,5271.427) |
|  | 2018 | 4377.481(3614.423,5225.159) |
|  | 2019 | 4318.792(3572.032,5189.857) |
|  | 2020 | 4178.907(3453.614,5033.216) |
|  | 2021 | 4084.214(3362.726,4960.088) |
| Americas | 1990 | 1909.691(1774.612,2061.114) |
|  | 1991 | 1902.665(1766.227,2056.125) |
|  | 1992 | 1882.513(1739.67,2042.735) |
|  | 1993 | 1879.507(1744.485,2028.401) |
|  | 1994 | 1881.303(1762.352,2023.065) |
|  | 1995 | 1899.346(1768.018,2040.181) |
|  | 1996 | 1930.522(1800.872,2070.102) |
|  | 1997 | 1935.836(1804.032,2077.613) |
|  | 1998 | 1907.746(1779.63,2051.43) |
|  | 1999 | 1878.405(1739.638,2021.306) |
|  | 2000 | 1867.857(1743.857,2010.077) |
|  | 2001 | 1858.424(1728.637,2013.304) |
|  | 2002 | 1817.703(1687.218,1971.508) |
|  | 2003 | 1798.36(1671.106,1946.715) |
|  | 2004 | 1788.563(1657.668,1932.44) |
|  | 2005 | 1752.395(1621.523,1906.017) |
|  | 2006 | 1713.225(1577.872,1863.485) |
|  | 2007 | 1696.483(1549.071,1860.136) |
|  | 2008 | 1665.203(1506.17,1828.091) |
|  | 2009 | 1636.304(1474.453,1802.095) |
|  | 2010 | 1578.865(1423.664,1745.287) |
|  | 2011 | 1556.887(1391.216,1722.162) |
|  | 2012 | 1514.627(1358.786,1685.302) |
|  | 2013 | 1466.784(1310.703,1639.665) |
|  | 2014 | 1426.73(1263.456,1605.289) |
|  | 2015 | 1391.193(1223.687,1573.846) |
|  | 2016 | 1359.289(1192.868,1555.585) |
|  | 2017 | 1330.64(1154.565,1542.697) |
|  | 2018 | 1297.789(1110.048,1522.262) |
|  | 2019 | 1266.662(1068.848,1501.127) |
|  | 2020 | 1188.549(982.854,1414.569) |
|  | 2021 | 1144.672(911.841,1396.03) |
| Eastern Mediterranean | 1990 | 1678.338(1348.988,2107.172) |
|  | 1991 | 1692.234(1365.41,2092.831) |
|  | 1992 | 1699.21(1375.373,2105.379) |
|  | 1993 | 1703.507(1367.238,2125.7) |
|  | 1994 | 1718.889(1387.893,2153.056) |
|  | 1995 | 1743.197(1407.317,2179.321) |
|  | 1996 | 1751.166(1437.177,2181.506) |
|  | 1997 | 1752.518(1427.128,2216.312) |
|  | 1998 | 1739.191(1421.427,2223.138) |
|  | 1999 | 1730.989(1416.063,2195.298) |
|  | 2000 | 1715.916(1389.077,2132.522) |
|  | 2001 | 1701.337(1377.683,2114.339) |
|  | 2002 | 1692.347(1384.423,2074.129) |
|  | 2003 | 1682.672(1379.302,2044.658) |
|  | 2004 | 1670.155(1378.314,2032.299) |
|  | 2005 | 1644.487(1361.238,1993.624) |
|  | 2006 | 1620.062(1351.431,1947.691) |
|  | 2007 | 1582.58(1315.217,1929.587) |
|  | 2008 | 1551.164(1289.18,1862.634) |
|  | 2009 | 1518.342(1261.849,1794.721) |
|  | 2010 | 1492.141(1236.539,1810.113) |
|  | 2011 | 1467.584(1207.683,1772.683) |
|  | 2012 | 1449.476(1174.246,1771.071) |
|  | 2013 | 1444.393(1162.279,1792.07) |
|  | 2014 | 1441.39(1138.803,1784.831) |
|  | 2015 | 1430.244(1121.701,1775.472) |
|  | 2016 | 1420.151(1106.751,1802.816) |
|  | 2017 | 1413.688(1094.09,1793.596) |
|  | 2018 | 1398.682(1077.219,1777.399) |
|  | 2019 | 1410.283(1090.244,1775.73) |
|  | 2020 | 1478.376(1151.947,1865.295) |
|  | 2021 | 1463.218(1132.559,1832.862) |
| European | 1990 | 613.137(536.228,704.467) |
|  | 1991 | 612.66(534.841,708.336) |
|  | 1992 | 597.973(517.558,697.576) |
|  | 1993 | 593.254(515.412,693.355) |
|  | 1994 | 589.351(510.28,682.708) |
|  | 1995 | 587.189(513.192,678.755) |
|  | 1996 | 579.239(508.744,666.203) |
|  | 1997 | 576.992(508.181,659.709) |
|  | 1998 | 574.995(509.268,656.613) |
|  | 1999 | 580.911(517.562,666.197) |
|  | 2000 | 572.91(511.909,658.821) |
|  | 2001 | 557.751(497.952,640.08) |
|  | 2002 | 536.317(480.033,612.054) |
|  | 2003 | 522.086(464.802,591.732) |
|  | 2004 | 501.599(449.55,562.916) |
|  | 2005 | 484.079(434.894,541.763) |
|  | 2006 | 471.93(423.47,525.655) |
|  | 2007 | 459.383(414.482,511.07) |
|  | 2008 | 450.208(408.241,495.877) |
|  | 2009 | 440.576(400.701,482.829) |
|  | 2010 | 425.211(388.09,466.329) |
|  | 2011 | 429.073(393.522,463.64) |
|  | 2012 | 442.128(407.631,477.353) |
|  | 2013 | 438.494(405.177,472.521) |
|  | 2014 | 437.895(403.34,471.944) |
|  | 2015 | 412.622(380.377,446.808) |
|  | 2016 | 399.445(366.598,435.585) |
|  | 2017 | 400.958(366.41,441.562) |
|  | 2018 | 395.398(358.015,438.606) |
|  | 2019 | 393.399(351.672,440.766) |
|  | 2020 | 368.715(323.048,419.596) |
|  | 2021 | 365.126(315.124,421.746) |
| Global | 1990 | 2648.993(2337.831,2953.867) |
|  | 1991 | 2666.652(2370.801,2980.161) |
|  | 1992 | 2677.359(2389.126,2988.839) |
|  | 1993 | 2689.467(2416.767,2991.774) |
|  | 1994 | 2700.621(2413.276,3016.667) |
|  | 1995 | 2712.579(2414.937,3036.434) |
|  | 1996 | 2721.63(2438.248,3041.987) |
|  | 1997 | 2721.39(2432.543,3028.904) |
|  | 1998 | 2721.245(2433.835,3023.274) |
|  | 1999 | 2716.224(2431.956,3029.758) |
|  | 2000 | 2696.396(2416.676,3015.945) |
|  | 2001 | 2673.168(2392.351,2995.854) |
|  | 2002 | 2649.637(2372.819,2973.335) |
|  | 2003 | 2634.863(2343.374,2970.974) |
|  | 2004 | 2619.49(2323.498,2966.967) |
|  | 2005 | 2591.684(2304.289,2925.569) |
|  | 2006 | 2539.023(2268.739,2876.366) |
|  | 2007 | 2489.79(2217.741,2817.885) |
|  | 2008 | 2442.145(2178.286,2749.993) |
|  | 2009 | 2414.578(2156.615,2690.649) |
|  | 2010 | 2393.218(2135.155,2686.803) |
|  | 2011 | 2374.03(2118.644,2664.3) |
|  | 2012 | 2337.026(2079.815,2632.178) |
|  | 2013 | 2314.659(2041.605,2605.66) |
|  | 2014 | 2286.416(1998.968,2584.913) |
|  | 2015 | 2235.674(1944.061,2547.111) |
|  | 2016 | 2185.925(1879.66,2513.814) |
|  | 2017 | 2161.138(1842.165,2497.88) |
|  | 2018 | 2147.899(1834.367,2478.659) |
|  | 2019 | 2164.054(1851.372,2521.769) |
|  | 2020 | 2142.933(1826.475,2508.86) |
|  | 2021 | 2118.49(1790.393,2490.714) |
| High SDI | 1990 | 726.371(643.723,813.796) |
|  | 1991 | 732.989(649.459,819.92) |
|  | 1992 | 729.892(645.651,821.957) |
|  | 1993 | 728.069(643.041,819.191) |
|  | 1994 | 723.657(637.114,814.411) |
|  | 1995 | 725.323(637.975,812.286) |
|  | 1996 | 722.476(642.039,809.068) |
|  | 1997 | 718.765(640.611,801.23) |
|  | 1998 | 713.534(642.525,793.783) |
|  | 1999 | 707.857(638.27,787.368) |
|  | 2000 | 686.113(621.82,766.971) |
|  | 2001 | 665.447(603.582,739.268) |
|  | 2002 | 653.083(591.278,724.156) |
|  | 2003 | 638.981(579.38,707.653) |
|  | 2004 | 608.749(550.69,677.075) |
|  | 2005 | 575.798(521.401,640.975) |
|  | 2006 | 544.721(493.787,599.613) |
|  | 2007 | 525.164(476.955,582.889) |
|  | 2008 | 514.48(467.97,568.96) |
|  | 2009 | 507.992(462.427,564.937) |
|  | 2010 | 500.12(456.979,557.026) |
|  | 2011 | 497.095(456.429,549.373) |
|  | 2012 | 488.196(446.384,540.599) |
|  | 2013 | 469.802(430.314,521.408) |
|  | 2014 | 462.878(424.509,506.682) |
|  | 2015 | 434.973(397.544,479.553) |
|  | 2016 | 414.913(377.447,456.912) |
|  | 2017 | 407.687(369.488,453.286) |
|  | 2018 | 399.05(359.414,445.436) |
|  | 2019 | 400.16(356.803,454.046) |
|  | 2020 | 382.445(332.046,439.318) |
|  | 2021 | 380.778(323.375,443.812) |
| High-middle SDI | 1990 | 342.067(306.926,383.415) |
|  | 1991 | 337.075(302.498,378.209) |
|  | 1992 | 329.644(295.723,368.879) |
|  | 1993 | 320.007(285.817,358.347) |
|  | 1994 | 319.655(286.259,355.728) |
|  | 1995 | 311.9(280.022,345.953) |
|  | 1996 | 303(272.476,335.412) |
|  | 1997 | 297.93(266.904,331.027) |
|  | 1998 | 292.749(261.452,324.801) |
|  | 1999 | 291.82(261.142,322.393) |
|  | 2000 | 288.905(259.365,319.24) |
|  | 2001 | 280.189(253.202,308.263) |
|  | 2002 | 277.043(250.675,303.264) |
|  | 2003 | 273.604(251.007,296.142) |
|  | 2004 | 267.876(247.83,287.608) |
|  | 2005 | 258.679(239.831,277.309) |
|  | 2006 | 244.159(227.841,260.718) |
|  | 2007 | 234.52(219.63,250.191) |
|  | 2008 | 220.473(207.702,233.47) |
|  | 2009 | 203.731(192.102,215.825) |
|  | 2010 | 189.532(177.953,199.859) |
|  | 2011 | 179.126(168.947,187.877) |
|  | 2012 | 175.478(165.918,184.847) |
|  | 2013 | 171.022(161.188,181.684) |
|  | 2014 | 170.146(160.896,181.129) |
|  | 2015 | 170.939(161.383,181.51) |
|  | 2016 | 170.179(159.911,181.259) |
|  | 2017 | 171.218(160.457,182.986) |
|  | 2018 | 169.11(157.775,181.036) |
|  | 2019 | 170.389(158.08,182.959) |
|  | 2020 | 161.756(147.698,175.473) |
|  | 2021 | 155.776(137.445,172.65) |
| Low SDI | 1990 | 3872.908(3343.756,4452.349) |
|  | 1991 | 3846.001(3326.348,4413.425) |
|  | 1992 | 3813.435(3327.984,4391.49) |
|  | 1993 | 3784.183(3289.018,4329.746) |
|  | 1994 | 3751.541(3219.348,4283.794) |
|  | 1995 | 3711.012(3214.329,4261.9) |
|  | 1996 | 3670.986(3185.36,4202.837) |
|  | 1997 | 3617.234(3155.157,4101.38) |
|  | 1998 | 3581.02(3163.356,4040.403) |
|  | 1999 | 3547.41(3116.183,3969.492) |
|  | 2000 | 3488.916(3085.776,3919.345) |
|  | 2001 | 3425.555(3046.936,3830.199) |
|  | 2002 | 3352.909(2981.529,3765.894) |
|  | 2003 | 3303.048(2918.282,3719.894) |
|  | 2004 | 3263.937(2865.58,3684.621) |
|  | 2005 | 3207.204(2820.228,3653.724) |
|  | 2006 | 3120.282(2762.819,3549.308) |
|  | 2007 | 3035.934(2678.256,3446.558) |
|  | 2008 | 2953.142(2594.528,3380.735) |
|  | 2009 | 2888.064(2514.007,3290.561) |
|  | 2010 | 2816.503(2462.848,3246.61) |
|  | 2011 | 2766.418(2399.385,3221.333) |
|  | 2012 | 2721.582(2340.358,3180.641) |
|  | 2013 | 2707.708(2333.418,3187.43) |
|  | 2014 | 2681.713(2302.114,3153.333) |
|  | 2015 | 2649.728(2257.633,3143.511) |
|  | 2016 | 2594.33(2191.121,3104.632) |
|  | 2017 | 2533.608(2115.053,3061.026) |
|  | 2018 | 2474.54(2042.147,2966.1) |
|  | 2019 | 2452.71(2014.127,2940.408) |
|  | 2020 | 2408.207(1970.11,2884.039) |
|  | 2021 | 2342.392(1899.171,2885.903) |
| Low-middle SDI | 1990 | 5361.85(4679.876,6127.373) |
|  | 1991 | 5324.364(4659.649,6114.983) |
|  | 1992 | 5265.681(4589.699,6033.646) |
|  | 1993 | 5210.84(4531.23,6012.441) |
|  | 1994 | 5168.513(4467.812,6005.646) |
|  | 1995 | 5121.486(4379.377,6013.948) |
|  | 1996 | 5059.148(4286.006,5986.446) |
|  | 1997 | 5004.163(4205.772,5943.428) |
|  | 1998 | 4970.718(4201.098,5888.658) |
|  | 1999 | 4924.685(4160.032,5830.672) |
|  | 2000 | 4873.502(4117.738,5789.544) |
|  | 2001 | 4828.29(4088.967,5768.052) |
|  | 2002 | 4796.544(4029.927,5778.669) |
|  | 2003 | 4765.66(4033.461,5794.594) |
|  | 2004 | 4742.117(4012.745,5763.802) |
|  | 2005 | 4726.678(4019.374,5734.816) |
|  | 2006 | 4674.278(3981.083,5692.712) |
|  | 2007 | 4627.303(3956.333,5624.945) |
|  | 2008 | 4566.061(3904.351,5530.78) |
|  | 2009 | 4515.872(3884.207,5449.146) |
|  | 2010 | 4467.37(3839.793,5395.151) |
|  | 2011 | 4443.932(3811.79,5382.829) |
|  | 2012 | 4429.784(3759.466,5372.701) |
|  | 2013 | 4410.809(3744.274,5282.51) |
|  | 2014 | 4379.402(3736.088,5232.832) |
|  | 2015 | 4323.856(3683.081,5119.691) |
|  | 2016 | 4255.718(3582.334,5033.156) |
|  | 2017 | 4160.008(3469.272,4953.832) |
|  | 2018 | 4075.186(3355.705,4922.235) |
|  | 2019 | 4021.8(3268.238,4919.597) |
|  | 2020 | 3911.049(3177.118,4785.782) |
|  | 2021 | 3819.39(3088.969,4708.461) |
| Middle SDI | 1990 | 1644.008(1417.887,1916.554) |
|  | 1991 | 1656.216(1434.923,1941.669) |
|  | 1992 | 1661.449(1448.727,1939.694) |
|  | 1993 | 1670.305(1452.418,1949.708) |
|  | 1994 | 1677.569(1470.552,1941.871) |
|  | 1995 | 1700.55(1491.751,1959.061) |
|  | 1996 | 1726.632(1521.673,1988.833) |
|  | 1997 | 1737.867(1550.312,1982.943) |
|  | 1998 | 1733.364(1550.397,1980.347) |
|  | 1999 | 1730.022(1545.673,1972.648) |
|  | 2000 | 1726.762(1546.647,1971.323) |
|  | 2001 | 1717.815(1536.772,1976.36) |
|  | 2002 | 1694.694(1517.6,1965.312) |
|  | 2003 | 1679.912(1497.144,1946.937) |
|  | 2004 | 1669.328(1473.349,1938.064) |
|  | 2005 | 1648.916(1457.665,1913.584) |
|  | 2006 | 1615.563(1437.251,1879.97) |
|  | 2007 | 1582.521(1402.93,1838.486) |
|  | 2008 | 1545.814(1366.146,1805.74) |
|  | 2009 | 1529.135(1338.065,1796.082) |
|  | 2010 | 1514.498(1324.94,1791.329) |
|  | 2011 | 1497.411(1308.323,1772.731) |
|  | 2012 | 1445.685(1252.367,1714.138) |
|  | 2013 | 1403.492(1193.114,1689.406) |
|  | 2014 | 1361.249(1157.604,1643.693) |
|  | 2015 | 1299.741(1102.075,1591.54) |
|  | 2016 | 1242.541(1049.875,1520.009) |
|  | 2017 | 1230.465(1033.004,1502.876) |
|  | 2018 | 1230.454(1017.762,1505.356) |
|  | 2019 | 1248.257(1022.273,1529.475) |
|  | 2020 | 1224.253(1021.66,1487.389) |
|  | 2021 | 1219.386(1006.514,1484.331) |
| South-East Asia | 1990 | 3610.178(3105.154,4203.041) |
|  | 1991 | 3588.019(3105.895,4170.31) |
|  | 1992 | 3555.175(3057.66,4128.773) |
|  | 1993 | 3521.219(3016.232,4074.768) |
|  | 1994 | 3478.746(2975.608,4012.655) |
|  | 1995 | 3422.822(2922.719,3977.703) |
|  | 1996 | 3362.373(2874.995,3888.426) |
|  | 1997 | 3289.113(2835.932,3776.042) |
|  | 1998 | 3250.807(2834.888,3707.268) |
|  | 1999 | 3213.409(2812.533,3664.05) |
|  | 2000 | 3145.6(2750.401,3603.29) |
|  | 2001 | 3078.212(2711.879,3540.888) |
|  | 2002 | 2998.186(2636.914,3421.66) |
|  | 2003 | 2940.809(2571.539,3392.192) |
|  | 2004 | 2902.86(2518.147,3370.766) |
|  | 2005 | 2850.795(2452.661,3348.367) |
|  | 2006 | 2738.033(2361.926,3238.461) |
|  | 2007 | 2640.896(2270.448,3086.907) |
|  | 2008 | 2541.972(2170.208,2963.193) |
|  | 2009 | 2488.622(2123.169,2913.268) |
|  | 2010 | 2428.201(2058.066,2833.888) |
|  | 2011 | 2389.363(2024.185,2798.216) |
|  | 2012 | 2349.183(1980.321,2794.551) |
|  | 2013 | 2337.677(1974.81,2790.047) |
|  | 2014 | 2307.955(1928.842,2739.4) |
|  | 2015 | 2285.115(1926.263,2774.113) |
|  | 2016 | 2221.635(1855.644,2673.147) |
|  | 2017 | 2173.033(1790.322,2620.795) |
|  | 2018 | 2130.411(1752.451,2603.918) |
|  | 2019 | 2115.338(1725.127,2561.226) |
|  | 2020 | 2067.596(1661.135,2507.083) |
|  | 2021 | 2006.665(1606.324,2449.72) |
| Western Pacific | 1990 | 883.966(748.822,1030.662) |
|  | 1991 | 890.8(764.37,1040.271) |
|  | 1992 | 899.188(775.105,1045.59) |
|  | 1993 | 903.659(779.634,1053.143) |
|  | 1994 | 901.95(785.887,1043.532) |
|  | 1995 | 907.63(784.383,1051.298) |
|  | 1996 | 915.587(797.988,1060.448) |
|  | 1997 | 917.014(800.105,1051.632) |
|  | 1998 | 908.597(791.99,1030.951) |
|  | 1999 | 908.338(794.836,1033.199) |
|  | 2000 | 905.965(788.306,1024.269) |
|  | 2001 | 894.981(769.488,1026.499) |
|  | 2002 | 883.857(767.5,1015.521) |
|  | 2003 | 875.093(766.235,993.376) |
|  | 2004 | 856.481(750.645,974.813) |
|  | 2005 | 834.333(725.777,951.534) |
|  | 2006 | 811.705(708.163,929.35) |
|  | 2007 | 790.771(688.422,902.575) |
|  | 2008 | 783.597(684.637,892.753) |
|  | 2009 | 788.525(691.161,897.614) |
|  | 2010 | 804.27(701.149,917.797) |
|  | 2011 | 794.511(699.539,916.453) |
|  | 2012 | 743.806(650.673,859.145) |
|  | 2013 | 710.1(614.146,813.985) |
|  | 2014 | 681.657(591.51,776.202) |
|  | 2015 | 629.673(542.28,719.454) |
|  | 2016 | 590.6(506.083,678.732) |
|  | 2017 | 586.918(496.729,684.617) |
|  | 2018 | 591.017(497.087,688.562) |
|  | 2019 | 620.586(520.492,733.07) |
|  | 2020 | 620.015(525.15,734.665) |
|  | 2021 | 635.234(533.709,750.179) |
| SDI: sociodemographic index; UI: uncertainty interval. | | |
